# Viral vectored vaccines: design, development, preventive and therapeutic applications in human diseases

**DOI:** 10.1038/s41392-023-01408-5

**Published:** 2023-04-07

**Authors:** Shen Wang, Bo Liang, Weiqi Wang, Ling Li, Na Feng, Yongkun Zhao, Tiecheng Wang, Feihu Yan, Songtao Yang, Xianzhu Xia

**Affiliations:** 1 Key Laboratory of Jilin Province for Zoonosis Prevention and Control, Changchun Veterinary Research Institute, Chinese Academy of Agricultural Sciences, Changchun, China; 2 College of Veterinary Medicine, Jilin University, Changchun, China; 3 China National Research Center for Exotic Animal Diseases, China Animal Health and Epidemiology Center, Qingdao, China

**Keywords:** Vaccines, Infectious diseases

## Abstract

Human diseases, particularly infectious diseases and cancers, pose unprecedented challenges to public health security and the global economy. The development and distribution of novel prophylactic and therapeutic vaccines are the prioritized countermeasures of human disease. Among all vaccine platforms, viral vector vaccines offer distinguished advantages and represent prominent choices for pathogens that have hampered control efforts based on conventional vaccine approaches. Currently, viral vector vaccines remain one of the best strategies for induction of robust humoral and cellular immunity against human diseases. Numerous viruses of different families and origins, including vesicular stomatitis virus, rabies virus, parainfluenza virus, measles virus, Newcastle disease virus, influenza virus, adenovirus and poxvirus, are deemed to be prominent viral vectors that differ in structural characteristics, design strategy, antigen presentation capability, immunogenicity and protective efficacy. This review summarized the overall profile of the design strategies, progress in advance and steps taken to address barriers to the deployment of these viral vector vaccines, simultaneously highlighting their potential for mucosal delivery, therapeutic application in cancer as well as other key aspects concerning the rational application of these viral vector vaccines. Appropriate and accurate technological advances in viral vector vaccines would consolidate their position as a leading approach to accelerate breakthroughs in novel vaccines and facilitate a rapid response to public health emergencies.

## Introduction

The outbreak of infectious diseases and the occurrence of cancers cause a huge impact on humans throughout history. Hemorrhagic fever, including Ebola, Marburg, and Lassa fever, cause fatality rates of up to 50%.^1–3^ In addition, there have been three waves of beta coronavirus emergence since 2003, of which coronavirus disease 2019 (COVID-19) has caused billions of confirmed cases and millions of deaths since 2019.^4–6^ Globally, an estimated 19.3 million new cancer cases and almost 10.0 million cancer deaths occur every year,^7^ which pose as the leading health threat.

For infectious diseases, vaccination and establishment of herd immunity are of primary importance. Among all vaccine technologies, recombinant viral vectors represent promising vaccine platforms due to their ability to express heterologous antigens and induction of cellular immune responses and humoral immune responses without exogenous adjuvants. Viral vector vaccines consist of viral particles whose genomes have been modified to contain one or more foreign genes encoding the targeted antigens. The rationale for using viruses to deliver the ‘vaccine gene’ is in several folds. Viral vectored vaccines are safe and induce both arm of innate and adaptive immune responses without involvement of the complete hazardous pathogen.^8^ Moreover, viral vectors have intrinsic adjuvant properties due to the expression of diverse pathogen-associated molecular patterns (PAMPs) and the activation of innate immunity.^9^ In addition, viral vectors can be engineered to deliver antigens to specific cells or tissues. Similarly, they can be rendered replication-competent or replication-deficient to increase their safety and reduce reactogenicity. Notably, the viral vector vaccine can recapitulate the natural infection process of specific pathogens, thus triggering classical acute inflammation and immune detection through the natural production of PAMPs, enabling mucosal delivery and induction of local-mucosal and systemic immunity. Several viral vector-based prophylactic vaccines have entered Phase III clinical trials or have been approved.^10–15^ In the field of cancers, viral vectors are ideal oncolytic viruses (OVs) since they can trigger cellular immunity and could be armed, shielded and targeting tumor cells. The release of tumorassociated antigens (TAAs) could activate and regulate the anti-tumor immune response. Several OV preparations have been approved for marketing, which present promising directions for immunotherapy of tumors.

Nevertheless, the systematic and comparative review of these viral vectors is less well established. Moreover, the generality and individuality of these viral vectors are not fully elucidated. In this review, the general overview of vesicular stomatitis virus (VSV), rabies virus (RABV), parainfluenza virus (PIV), measles virus (MeV), Newcastle disease virus (NDV), influenza virus (IFV), adenovirus (AdV), and poxvirus vector vaccines was summarized in terms of their application to life-threatening infectious diseases as well as immunotherapy for cancer. The characteristics, merits and limitations of these viral vectors were analyzed and presented in depth. Taken together, these issues would compel the acceleration and approval of novel viral vector vaccines confronting human health threats.

### Structure and design strategies for viral vectors

#### Nonsegmented negative‐strand RNA viruses (NNSVs) as vaccine vectors

**Fig. 1:**
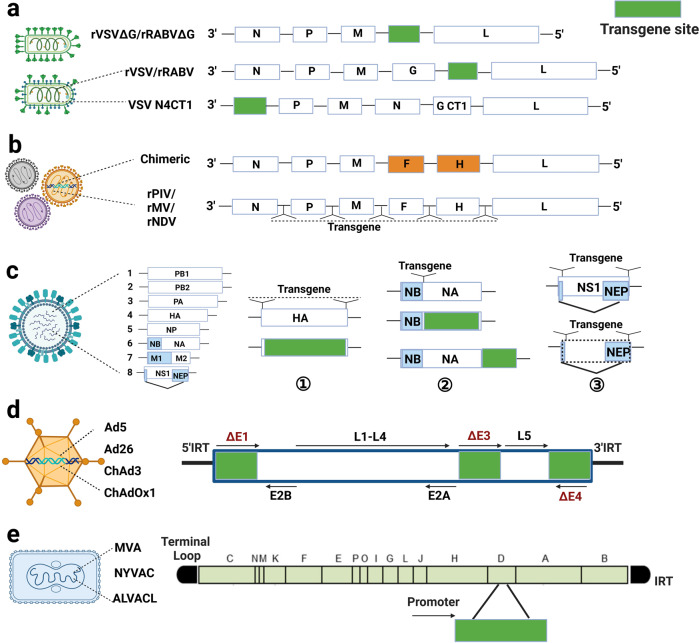
Design strategies for Vesicular stomatitis virus (VSV), rabies virus (RABV), parainfluenza virus (PIV), measles virus (MeV), Newcastle disease virus (NDV), influenza virus (IFV), Adenovirus (AdV) and poxvirus for vaccine platforms. **a** rVSVΔG/rRABVΔG, in which the glycoprotein (G) of the vector is replaced by a foreign gene; rVSV/rRABV, an additional transcription unit is involved between G and L of the genome. N4CT1, involves an additional transcriptional unit at the 3′ end of the genome, translocation of N gene and truncation of the VSV G cytoplasmic tail. **b** Chimeric paramyxovirus vector, in which the fusion and hemagglutinin glycoprotein of paramyxovirus is replaced by those of other paramyxoviruses; recombinant paramyxovirus vectors, which involve an additional transcription unit for foreign genes. **c** Manipulation of the genome of IFV based on HA, NA, and NS. c1. Inserting foreign gene based on HA: the foreign gene is inserted into the receptor binding site of the HA head or the N-terminal of HA; insert the foreign gene in place of HA while retaining packaging sequences. c2. Inserting foreign gene based on NA: the foreign gene is inserted into the stem of NA; preserve the non-coding sequences and adjacent coding regions of NA for transgene in place of NA coding sequence; involves an additional transcription unit at the 5′ end of NA. c3. Inserting foreign gene based on NS: insert transgene after the 125th amino acid of NS; retain NS1 and NEP, and introduce 2 A self-cutting site at the end of NS1; insert transgene in place of NS1. **d** Genome of AdV, E1, E3 and (or) E4 regions are designed for transgene. **e** Genome of poxvirus, the D transcription units could be replaced by the transgene of choice under the promoter. (Created in BioRender)

VSV and RABV are enveloped NNSVs belonging to Rhabdoviridae. Rhabdoviridae is composed of five structural proteins including nuclear protein (N), phosphoprotein (P), matrix protein (M), glycoprotein (G), and RNA-dependent RNA polymerase (L).^16,17^ PIV, MeV and NDV belong to Paramyxoviridae. Their nucleotide genome contains six structural genes including N, P, M, fusion glycoprotein (F), hemagglutinin glycoprotein (H), and L.^18,19^ Both surface envelope glycoproteins are responsible for host cell binding and invasion. The rescue and operation of these NNSVs were accomplished through reverse genetics approaches of negative single strand RNA. In 1994, RABV was the first to be rescued from cloned cDNA, which marking a major milestone in the field of NNSVs.^20^ The virus was rescued from a cloned cDNA that contains the full genome sequence in the positive‐sense orientation flanked by a T7 promoter and hepatitis delta virus ribozyme. Subsequently, the reverse genetic system of other NNSVs was established, which enables the reconstruction of the full-length genome.^21–30^ For these NNSV vectors, there are two major strategies for foreign gene delivery. (1) Delete the glycoprotein gene of the viral vector and replace it with a targeted gene (NNSVΔG or NNSVΔF) (Fig. 1a, b).^31^ (2) Involving an additional transcriptional unit for foreign antigen while retain the vector glycoprotein gene in the full-length genome (rNSSV) (Fig. 1a, b).^32–34^ Foreign genes could be inserted at different gene junctions of the genome as an additional expression cassette.

In the NNSVΔG/NNSVΔF design strategy, the targeted glycoprotein could be displayed on the surface of the recombinant virus. Accordingly, the cell and tissue tropism of the recombinant virus is largely depended on foreign glycoproteins. In cases that the target glycoprotein was similar in the molecular size and function of the vector glycoprotein, NNSVΔG/NNSVΔF design strategy rendered the recombinant virus ideal for biological growth properties and minimization of anti-vector immunity.^35,36^ Although recombination of large foreign genes is achievable, the growth titer of recombinant virus is relatively low. For example, rVSVΔG-SARS-CoV-2-S and rVSVΔG-CCHFV-G represented an upmost growth titer of about 10^6^ TCID_50_.^37,38^ To overcome this issue, truncation of the cytoplasmic tail (CT) region of the foreign gene and screening of the optimum cell line for virus culturing are alternative measures. In contrast, a higher growth titer could be achieved in viral vectors that carries an additional transcriptional unit for the external gene.^32,39^ Foreign genes other than glycoprotein can also be incorporated into recombinant viruses. In some cases, transmembrane (TM) and CT domains of the foreign gene should be replaced by those of the glycoprotein of the viral vector to maximize the incorporation of the foreign protein into the virion and optimize immunogenicity.^40^ Of particularly note, transcriptional translation decreased from 3′ to 5′ end of the genome.^41^ For example, the polar mechanism of VSV transcription results in a gradient of mRNA abundance that is highest at the 3′ end of the genome and decreases toward the 5′ end, following the order of N > P > M > G > L, thus the expression level of specific antigens was correlated with the insertion position. An ideal insertion site for the foreign gene should balance virus replication and foreign gene expression and contain an optimized arrangement of gene junction sequences before and after the exotic gene.^42–44^

#### Segmented RNA (IFVs) as vaccine vectors

IFV is an enveloped, segmented RNA virus belonging to the Orthomyxoviridae family.^45^ IFV is classified into four genera according to nucleoprotein (NP): influenza A, B, C, and D. Of which influenza A virus (IAV) and influenza B virus (IBV) viruses are of public health relevance due to their potential to cause severe disease in humans. IAV and IBV carry 8 segments of single-stranded, negative-sense RNA that encode at least 8 proteins: polymerase basic 1 (PB1), polymerase basic 2 (PB2), polymerase acidic (PA), hemagglutinin (HA, surface glycoprotein), NP, neuraminidase (NA, surface glycoprotein), NB (surface glycoprotein), matrix protein 1 (M1 and M2), non-structural protein (NS1 and NEP). Based on reverse genetic approaches of IFV,^46–48^ multiple segments of IFV were manipulated for transgene, including HA, NA, NS1, etc. (Fig. 1c), chimeric construction between IAV and IBV was also reported.^49–56^

When a foreign gene was inserted into the receptor binding site of HA head or the N-terminal of HA, the function of IFV HA was not affected, thus complete replication ability retained. In the case of the construction of replication-defective recombinant virus, only the packaging sequences of the 3′ and 5′ ends of HA were retained, and the coding region of HA was replaced by foreign sequences. This replication-defective virus could replicate in Madin-Darby canine kidney (MDCK) cell lines that stably express HA protein.

For NA stem, only 28–41 amino acid insertion is permissive. Inserting foreign sequences into the NA stem would affect the virulence of the virus.^57,58^ Another strategy concerning the NA fusion proteins was prepared by preserving the non-coding sequences and adjacent coding regions of NA. In this strategy, IFV was mostly replication defective, which required the addition of exogenous NA enzymes. There is also a strategy that involved an additional transcription unit at the 5′ end of IFV NA, which maintained the complete structure and function of NA. Approximately 680 bp foreign gene fragments were allowed.^59^ Overall, ~1.5 kb of the foreign gene was permissive to be incorporated into the IFV NA segment.^60^

The nonstructural protein 1 (NS1) and nuclear export protein (NEP) are encoded by the NS gene of IFV, which can tolerate 250 amino acids insertion. NS1 protein of IFV is a virulence element which could inhibit the interferon production and lead to the escape of the IFV to the initial immune response.^61^ The deletion of NS1 gene weakened the virulence of the virus significantly, which has been applied to the development of IFV-vectored vaccine.^62^ NEP works in regulating the IFV ribonucleoprotein complex and virus nucleation. NS is not involved in virion formation, thus NS protein change does not alter the antigenicity of IFV.^63^ There are three methods to construct chimeric IFV vector vaccines based on NS segments. The first construction method is to establish a bicistronic reading frame, that is, inserted a start-stop reading frame (UAAUG) after the 125th amino acid.^63^ The second construction method retained NS1 and NEP, and introduced 2 A self-cutting site at the end of NS1.^64^ Finally, in the case of NS1 deletion constructs, the replication ability of the recombinant virus in MDCK cells was significantly weakened. To address this issue, mutations in M gene A14U enhanced replication of NS1-deleted viruses in MDCK cells.^65^ Indeed, NS1 gene deletion may not merely act as an attenuation strategy, but exhibit more potent and long-lasting immunity compared to cold-adapted IFV by activating multidimensional immune responses.

#### Adenoviruses as vaccine vectors

AdVs are non-enveloped dsDNA viruses belonging to Adenoviridae.^66^ AdVs are of wide host origin and can be divided into various serotypes. Their double-stranded linear genome ranges from 26 kb to 45 kb, a size that is amenable to manipulation.^67^ AdVs have transition from tools for gene replacement therapy to bona fide vaccine delivery vehicles. They are attractive vaccine vectors as they simulaneously induce both innate and adaptive immune responses in mammalian hosts. AdV-based vectors can be rendered replication-competent or replication-defective via the manipulation of early 1 (E1) region or part of it.^68^ In addition, the early 3 (E3) gene could be deleted to enlarge the capacity for transgene insertion since the E3 gene is dispensable for virus replication. Consequently, E1 or E3 deleted regions are expression cassettes for transgene expression (Fig. 1d). AdV vectors are well established, easy to operated, amenable to rapid, inexpensive manufacturing and cold chain-free storage. AdVs of human, simian and avian origin are involved in vaccine vectors.

#### Poxviruses as vaccine vectors

Poxvirus is the largest enveloped DNA virus. In the 1980s, smallpox was successfully eradicated by vaccination with the vaccinia virus (VACV). During the same period, VACA was applied as a transgenic expression vector.^69,70^ The passage of parental VACA resulted in random mutations and deletions, which contributed to the reduced pathogenicity of VACV. The third generation poxvirus vectors include Listeria clone 16m8 (LC16m8), Dairen I strain (Dis), M65, M101, modified vaccinia virus Ankara (MVA) as well as several attenuated fowlpox viruses.^71^

MVA is highly attenuated by passaging 570 generations on chicken embryos. Due to the blocking in virus assembly, MVA doesn’t produce infectious progeny while maintains robust DNA replication and antigen expression ability in most mammalian cells.^72–74^ Thereinto, MVA-572, MVA-I721 and MVA-BN share 100% identical nucleotide sequence in coding regions while exhibit significantly different phenotypes. Among them, MVA-BN shows better safety and immunogenicity than other two strains.^75^ MVA is an excellent third-generation smallpox vaccine that has been vaccinated by more than 120,000 people in Germany.^71,76–79^ MVA-VLP HIV vaccine candidate has shown excellent safety in clinical trials of 500 people, including immunocompromised individuals and HIV patients.^80,81^ Recombinant MVA is genetically stable, easily modified, safe and shows good immunogenicity even under the preexisting anti-vector immunity, especially when used in combination with other viral vector vaccines, such as AdV vector vaccine.^82–85^ These characteristics make MVA a promising vaccine vector. In addition to MVA, other poxviruses are used as vectors including Canarypox virus (ALVACL), C16m8 deriving from the Lister strain as well as New York attenuated vaccinia virus NYVAC (Fig. 1e). Comparison of viral vectors was summarized in Table 1.

**Table 1:** Comparison of viral vectors

Vector	Type of virus(kb)	Genome size(kb)	Genome type	Cargo capacity(kb)	Predominant immune response	Administration route	Strengths	Weaknesses	References
Vesicular stomatitis virus	Enveloped, RNA	~11	Single stranded, negative‐sense, nonsegmented	~6	Humoral and cellular immune response	IM, IN, or OR	No concerns of virulence reversion, residual virulence or virus recombination; small and easily manipulated genome; stable expression of foreign genes; rapid replication and high growth titer	Safety concerns	^514^
Rabies virus	Enveloped, RNA	~12	Single stranded, negative‐sense, nonsegmented	~6.5	Humoral response in dominant	IM or OR	Small and easily manipulated genome; design as inactivated bivalent vaccines	A potential risk for reversion to virulence; less well immunogenicity than VSV vector	^515,516^
Parainfluenza virus	Enveloped, RNA	~15	Single-stranded negative-sense, nonsegmented	~4	Humoral, cellular and mucosal immune response	IM, IN, or OR	Ideal for paediatric and respiratory diseases; safe; genomic stability	Anti-vector immunity; Safety concerns	^44^
Measles virus	Enveloped, RNA	~16	Single-stranded negative-sense, nonsegmented	~6	Humoral, cellular and mucosal immune response	IM, IP or SC	Licensed live-attenuated measles vaccines are effective and safe; lack of genomic integration in the host; established manufacturing infrastructure	Limited challenge models; low viral titers	^378,517–519^
Newcastle disease virus	Enveloped, RNA	~15	Single-stranded negative-sense, nonsegmented	~4	Humoral and cellular immune response	IM,IN	High growth titers; lack of genomic integration in the host; host restriction; no pre-existing antibody to NDV in the human	Less well immunogenic than other paramyxovirus vector-based vaccines	^210^
Lentivirus	Enveloped, RNA	~9.2	Single-stranded positive-sense, nonsegmented	~4	Humoral and cellular immune response	IM,IN	Low anti-vector immunity; less integration into the host genome; Durable immune responses	Safety concerns; potential batch to batch variation in manufacturing	^8^
Influenza virus	Enveloped, RNA	~13.5(total), 0.89–2.3 kb per each segment	Single stranded, negative‐sense, segmented	<1.5	Humoral and cellular immune response	IM, IN	A broad host range; easily manipulated genome; highly attenuated; established manufacturing infrastructure	Limited transgene ability; genetic reassortment; safety concerns	^520–522^
Adenovirus	Non-enveloped, DNA	26–45	Double-stranded, nonsegmented	~7.5	Humoral and cellular immune response	IM, IN, or OR	Well-established; high transduction efficiencies; relative large capacities for transgenes; high titer of production	Anti-vector immunity	^523^
Poxvirus	Enveloped, DNA	130–300	Double-stranded, nonsegmented	~25	Low/moderate antibodies response and strong cellular immune response	IM	Packing flexibility of the genome; without genomic integration in the host; expressing VLPs	Existence of the viral immunomodulatory genes	^8,524^

*IM* intramuscular, *IN* intranasal, *OR* oral, *IP* intraperitoneal, *SC* subcutaneous, *VLPs* virus like particles, *VSV* Vesicular stomatitis virus

### Application of viral vector vaccines in human disease

#### Vesicular stomatitis virus vector

##### A single dose of VSV-vectored vaccine is potent in inducing long-lasting protection

In most cases, VSV vectored vaccines are designed as a single dose regime. For viral hemorrhagic fever, a single dose of VSV vectored vaccine induced long-lasting protection. Representatively, rVSVΔG-ZEBOV, a recombinant EBOV vaccine candidate in which VSV G gene was replaced with the G gene of Zaire Ebola virus (ZEBOV) for the rescue of recombinant virus. A single intramuscular (IM) dose vaccination of rVSVΔG-ZEBOV fully protected mice and non-human primates (NHPs) against the lethal challenge of EBOV.^86–91^ Animals with delayed activation of innate responses succumbed to challenge.^92^ In Guinea ring vaccination, a single dose vaccination of 2 × 10^7^ PFU of rVSVΔG-ZEBOV showed good safety and immunogenicity in volunteers. rVSVΔG-ZEBOV offered substantial protection against EBOV disease, with an overall protective efficacy of 100%.^93^ After vaccination, antibodies appeared on day 14, peaked around day 28, and were detectable within 2 years.^94–96^ rVSVΔG-ZEBOV has been approved by the European Medicines Agency (EMA) and has been licensed for emergency use.^10,97,98^

Similarly, a single dose vaccination of rVSVΔG vectored vaccine expressing the glycoprotein of other haemorrhagic fever viruses like Marburg virus (MARV), Lassa virus (LASV) and Crimean-Congo hemorrhagic fever virus (CCHFV) protected NHPs completely.^38,87,99,100^ For MARV vaccine candidate, a recombinant VSV-based virus expressing MARV (Musoke strain) GP (rVSVΔG-MARV-GP) showed cross-protection against MARV Angola and Ravn strain in NHPs.^101^ rVSVΔG-MARV-GP vaccinated cynomolgus monkeys were challenged ~14 months after vaccination, no clinical signs of disease were observed in vaccinated animals. In outbred guinea pigs, a single dose of VSV-based recombinant virus expressing LASV GP (rVSVΔG/LASV-GPC) induced rapid and long-term protection.^102^ Protection rates at 25 days, 6 months and 1 year post vaccination were 83%, 87% and 71%, respectively. For CCHFV, a single dose of rVSVΔG-vectored vaccine expressing CCHFV glycoprotein precursor (GPC) showed good tolerability and achieved 100% protection against the lethal challenge of CCHFV in mice.^38^

American Hantavirus Cardiopulmonary Syndrome (HCPS) is caused by Andes virus (ANDV) and Sinobrei virus (SNV). Prescott, J. et al. constructed a rVSVΔG-vectored vaccine rVSVΔG/ANDVGPC in which the GP of VSV was replaced by ANDV GPC.^103^ A single IM dose vaccination of rVSVΔG/ANDVGPC induced high titers of NAbs and achieved sterile immunity in hamsters. The post-challenge protective efficacy was 100%. In another study, the vaccine was effective against ANDV infection 6 months after inoculation in hamsters whilst no protective efficacy was observed 1 year after inoculation. Warner, BM et al. constructed two live vector vaccines, rVSVΔG/SNVGPC and rVSVΔG/ANDVGPC, which expressed GPC of SNV and ADNV, respectively.^104^ Both rVSVΔG/SNVGPC and rVSVΔG/ANDVGPC induced a cross-reactive immune response and played a protective role in Syrian hamsters.

Similarly, a single does vaccination of VSV-based vaccine expressing surface glycoprotein of other pathogenetic viruses, such as Nipah virus (NiV),^105–107^ Zika virus (ZIKV),^108–111^ severe acute respiratory syndrome coronavirus 1 (SARS-CoV-1)^112,113^ and Middle East respiratory syndrome coronavirus (MERS-CoV),^114^ proved to be immunogenic and protective in preclinical animal models. The above studies emphasized that VSV vectored vaccines could completely protect against a large part of pathogens post a single IM dose of injection, and the immune response is durable, which represents the prominent feature of VSV vector vaccine.

##### Multivalent VSV-vectored vaccines protect animals from lethal challenges of multiple pathogens

Multivalent vaccines are of great significance in areas where multiple severe pathogens overlap, such as West Africa. According to previous research, rVSVΔG strategy exhibited weakened neurovirulence and experienced lower anti-vector immunity.^115–120^ Moreover, rVSV vectored vaccines expressing different foreign proteins could be inoculated simultaneously without interference of post-challenge protection of all targeted pathogens.^121^ These results enlightened the potential of VSV vectored vaccines for multivalent administration. In a preclinical study, a single dose vaccination of a recombinant bivalent vaccine VSVΔG/DUAL expressing ZEBOV and ANDV glycoproteins achieved sterile immunity to ZEBOV and ANDV in hamsters.^122^ Geisbert, T. W. et al. conducted a multivalent vaccine involving Sudan Ebola virus (SUDV), ZEBOV, Cote d’Ivoire Ebola virus (CIEBOV) and MARV.^123^ Cynomolgus monkeys were vaccinated with the multivalent vaccine consisting of equal doses of VSVΔG/SUDV GP, VSVΔG/ZEBOV GP and VSV ΔG/MARV GP. When challenged with the above four filoviruses, all vaccinated macaques survived. Likewise, the tetravalent VSV-vectored vaccine expressing antigens from LASV, EBOV, MARV and SUDV achieved 100% protection against the four hemorrhagic fever viruses including LASV, EBOV, MARV and SUDV after two doses.^124^ NAbs to the glycoproteins of the four filoviruses were detected in all vaccinated animals, while cell-mediated immune responses to glycoproteins were also detected in most vaccinated cynomolgus monkeys. rVSV-N4CT1 vector was also applied in trivalent vaccine development against EBOV, SUDV, and MARV.^124^ Although the trivalent vaccine exhibited decreased immunogenicity compared to the monovalent vaccine, the protective effect remained at 100%. The above results suggest that VSV-based monovalent vaccine are applicable. Representative VSV vector-based vaccines for human disease were summarized in Table 2.

**Table 2:** Vaccine candidates based on vesicular stomatitis virus vector

Pathogen	Design strategy	Stage	Results	Advantages	Overall concerns	Reference
Ebola virus	rVSVΔG-EBOV GP	Phase III	100% protection	Postexposure, long-term, and cross protection; single dose regimen	Safety concerns, adverse effect	^93,525,526^
Marburg virus	rVSVΔG-MARV GP	NHPs	100% protection	Sterile immunity; single-dose	Safety concerns	^87,99,101,527–529^
Lassa virus	rVSVΔG-LASV GPC	NHPs	100% protection	Long-term, cross-protection; multivalent; single-dose	Safety concerns	^100,121,530^
CCHFV	rVSVΔG-CCHFV GPC	Mice	100% protection	Stronger immunogenicity than RABV-based CCHFV vaccine candidates	Safety concerns	^38,125^
Andes virus	rVSVΔG-ANDV GP	Hamsters	100% protection	Postexposure protection; cross-protection; sterile immunity	Safety concerns	^103,104^
SARS-CoV	rVSV-S/rVSVΔG-S	Mice	/	Long-term antibody response	Safety concerns	^112^
MERS-CoV	rVSVΔG-S	NHPs	/	Long-term antibody response	Safety concerns	^114^
SARS-CoV-2	rVSVΔG-S	Phase I	/	Reduce viral load; mucosal delivery	Poor immunogenicity post IM vaccination	^332^
Nipah virus	rVSVΔG-NIV F/G/F + G	NHPs	100% protection	Single round replication	\	^107^
Hendra virus	rVSV-HEV G	Mice	/	More immunogenic than RABV vector-based vaccine candidate	Safety concerns	^126^
Zika virus	rVSV-prM-E-NS1	Mice	100% protection	MTase-defective, co-expression of prM and E, higher levels of Th2 and Th17 cytokine responses	Safety concerns	^111,531,532^

*CCHFV* Crimean Congo hemorrhagic fever virus, *SARS-CoV* severe acute respiratory syndrome coronavirus, *MERS-CoV* Middle East respiratory syndrome coronavirus, *SARS-CoV-2* severe acute respiratory syndrome coronavirus 2, *NHPs* nonhuman primates, *IM* intramuscular, *RABV* rabies virus, *MTase* methyltransferase, *prM* membrane precursor, *E* envelope

#### Rabies virus vector

##### Inactivated RABV-vectored vaccines combined with adjuvant confer full protection and trigger long-lasting immune responses

Although live RABV could be attenuated through genetically engineered strategies, a live recombinant RABV is unlikely to be approved due to safety concerns. Simultaneously, attenuated and replication-defective RABV vector vaccines were less immunogenic compared to VSV vectored vaccines expressing homologous antigen.^125–127^ Alternatively, inactivated RABV-vectored vaccines were safe and immunogenic, which represented a reasonable choice.^128–130^

For viral hemorrhagic fever, replication-competent and replication-defective vaccine candidates expressing ZEBOV GP were generated based on RABV BNSP333 vector.^131^ ZEBOV GP proteins could be efficiently incorporated into virions. Immunization with a live or inactivated vaccine candidate induced humoral immunity and conferred protection against both lethal RABV and EBOV challenges in mouse models. Further evaluation in NHPs showed that the replication-competent vaccine conferred 100% protection against EBOV infection, while the replication-defective or inactivated vaccine provided only 50% protection.^132^ Improvements were made to overcome the unsatisfactory protective efficacy of the inactivated vaccine by increasing the amount of GP incorporation into RABV virions through GP codon optimization.^133^ After that, two or three doses of BNSP333-coZGP (FILORAB1) adjuvanted with GLA-SE induced robust ZEBOV GP-specific IgG, NAbs and provided 100% protection after the lethal challenge of EBOV in NHPs.^134^

Meanwhile, SUDV and MARV vaccines have been developed based on the same strategy. FILORAB3 is a MARV vaccine expressing a codon-optimized GP of MARV Angola strain based on the RABV BSNP333 vector.^128^ Inactivated FILORAB3 adjuvant with Toll-like receptor 4 (TLR-4) agonist (GLA-SE) induced potent MARV GP-specific IgG antibodies. Interestingly, mice in the live FILORAB3 vaccination group succumbed to lethal challenge, while a single dose of inactivated FILORAB3 adjuvanted with GLA-SE conferred full protection. NK cell-dependent antibody-mediated cellular cytotoxicity (ADCC) played a critical role in immune protection in mice, which was consistent with the protective mechanism of RABV-vectored LASV vaccine.^129^ RABV vector has also been widely utilized to in vaccine development for genome-segmented pathogens, such as LASV and Rift Valley fever virus (RVFV). LASSARAB was a bivalent vaccine candidate that expressed codon-optimized LASV GPC based on BNSP333.^129^ Inactivated LASSARAB adjuvanted by GLA-SE induced long-lasting humoral responses to LASV and RABV in mice and guinea pigs. LASSARAB fully protected guinea pigs and mice against the LASV challenge mainly through non-NAbs-mediated ADCC and antibody-dependent cell-mediated phagocytosis (ADCP). Our group expressed codon-optimized RVFV eGn glycoprotein based on the RABV SRV9 strain, termed rSRV9-eGn.^135,136^ Inactivated rSRV9-eGn combined with poly (I:C) and ISA201VG adjuvant induced cellular immune response and RVFV-specific IgG antibodies. Moreover, rSRV9-eGn immunized mice produced memory T cell-dominant proliferating T cells.

Inactivated RABV-vectored vaccines also exhibit efficacy in emerging beta coronavirus.^137,138^ Full-length S protein incorporation into the RABV vector reduced the growth titers of recombinant virus.^139^ Thus BNSP333-S1 was constructed, which contains the MERS-CoV S1 domain that fused with the C-terminus of RABV G protein.^139–144^ Inactivated BNSP333-S1 induced high levels of NAbs in mice and conferred complete protection against the fatal challenge of MERS-CoV. In our previous study, a parallel comparison was conducted between recombinant RABV SRV9 vectored vaccine candidate expressing MERS-CoV S1 protein fragment and Gram-positive enhancer matrix (GEM) particles displaying MERS-CoV receptor binding domain (RBD) protein.^145^ The RABV vector-based vaccine induced remarkably earlier antibody response and higher levels of cellular immunity, while the GEM particle vector-based vaccine induced a higher antibody response, even at a low dose of 1 µg. This study described a platform-dependent manner of MERS vaccines. CORAVAX is an inactivated RABV SADB19 vectored COVID-19 vaccine candidate expressing S1 of severe acute respiratory syndrome coronavirus 2 (SARS-CoV-2) spike (S).^146–148^ A single dose of CORAVAX vaccine induced high levels of SARS-CoV-2 and RABV NAbs, yet two doses were required for complete viral clearance in the nasal turbinate. CORAVAX was highly effective and conferred protection against hamster model post SARS-CoV-2 challenge. TLR4 agonist (AddaVax) was determined to have the greatest potential according to quality antibody titers. Pre-existing RABV immunity showed no significant impact on the immune response. Antigen-specific serum antibody titers and long-lived antibody-secreting cells in the spleen and bone marrow lasted over 1-year post CORAVAX immunization.^149^ Human clinical trials of CORAVAX are ongoing. Our group developed inactivated recombinant viral vector vaccines based on the RABV SRV9 strain, which chimerically expressed RBD or S1 of SARS-CoV-2 in the additional transcriptional unit of RABV genome.^150^ Combined with poly(I:C) and ISA 201VG adjuvant, three dose of inactivated recombinant viruses (SRV-nCoV-RBD or SRV-nCoV-S1) induced durable NAbs against SARS-CoV-2 and RABV. Notably, inactivated SRV-nCoV-RBD induced earlier and well-maintained antibody production than SRV-nCoV-S1. In further evaluations, inactivated SRV-nCoV-RBD induced NAbs against both SARS-CoV-2 and RABV in cats and dogs, with a relatively broad-spectrum cross-neutralization capability against SARS-CoV-2 variants of concern (VOCs).

For encephalitis viruses, a recombinant NIV vaccine expressing NiV G was constructed based on BNSP333, termed NIPARAB. After intranasal (IN) inoculation with live NIPARAB, mice showed no clinical signs of disease.^130^ Although mice intramuscularly inoculated with a single dose of live NIPARAB or two doses of inactivated NIPARAB produced NAbs and NIV-G-specific binding antibodies, a higher antibody level was only observed in the inactivated vaccine group. Of note, anti-NIV G-specific immune serum had cross-reactivity against Hendra virus (HEV), another paramyxovirus that causes fatal encephalitis. Parallel comparisons between VSV and RABV-based HEV vaccine were conducted^126^ Codon optimization increased the incorporation of HEV G into the RABV BNSP333 vector by 2–3 times, while it had no influence on the VSV-vectored vaccine candidate compared to those expressing the original antigen sequence. Surprisingly, both vaccine candidates were safe and induced high levels of HEV G-specific antibodies in mice. Three doses of inactivated vaccines induced higher levels of HEV G-specific IgG and NAbs than that of a single dose of live vaccine. Under the same conditions, the VSV-vectored live vaccine induced higher HEV G-specific antibodies and NAbs than the RABV vector live vaccine, which might be due to the rapid replication ability of VSV. Overall, considering the biosafety issue and the lower immunogenetics of RABV compared to VSV-based vaccines, inactivated form seems to be a more attractive direction. Representative RABV vector-based vaccines for human disease were summarized in Table 3.

**Table 3:** Vaccine candidates based on rabies virus vector

Pathogens	Design strategy	Stage	Results	Advantages	Overall concerns	Reference
Ebola virus	BNSP333-GP	NHPs	100% protection	\	Poor NAbs; safety concern	^131,132,458,533^
	INACBNSP333-GP	NHPs	50% protection	Safe	Poor NAbs	^133^
	INACBNSP333 co (EBOV + SUDV + MARV) GP	NHPs	100% protection	Safe; immunogenic; high titer of NAbs	\	^134^
	rERAG333E-(EBOV + SUDV) GP	Dogs	NAbs and specific Abs	Long-term protection (1 year); oral delivery	Safety concern	^335,336^
Marburg virus	INACBNSP333-coGPC	Mice	100% protection	Safe	Poor NAbs	^128^
Lassa virus	BNSP333-coGPC	Guinea pigs	40% protection	\	Poor binding IgGs	^129^
	BNSPΔG-coGPC	Mice	\	\	Poor binding IgGs	
	INACBNSP333-coGPC	Guinea pigs	80% protection	Safe	No NAbs	
RVFV	rSRV9-eGn	Mice	\	Safe	Poor NAbs	^135,136^
MERS-CoV	INACBNSP333-S1	Mice	100% protection	High titer of NAbs; safe	\	^139^
	RVΔP-S1	Mice	NAbs	Safe	\	^534^
	INACrSRV9-S1	Mice	\	Earlier humoral and cellular immunity	\	^145^
SARS-CoV	pSPBN-333-S	Mice	Binding Abs and NAbs	\	\	^137^
SARS-CoV-2	BNSP333-S1	Golden hamsters	NAbs and reduced virus load	Single dose; safe; long-lasting immune response	\	^146,147,149^
	rSRV9-RBD/S1	Mice, cats and dogs	NAbs against SARS-CoV-2 and RABV	Long-lasting antibody response (4 months); broad-spectrum immune response	\	^150^
Nipah virus	INACBNSP333-G	Mice	G-specific Abs and NAbs	Cross-protection	\	^130^
	BNSP333-G	Mice	G-specific Abs and NAbs	\	\	
	rERAG333E-G/F	Mice and Pigs	G/F-specific Abs and NAbs	Oral delivery	\	^338^
Hendra virus	BNSP333-coG	Mice	G-specific Abs and NAbs	\	Poor G-specific Abs	^126^
	INACBNSP333-coG	Mice	G-specific Abs and NAbs	More immunogenic than RABV vector-based live vaccines	\	

*RVFV* Rift Valley fever virus, *MERS-CoV* Middle East respiratory syndrome coronavirus, *SARS-CoV* severe acute respiratory syndrome coronavirus, *SARS-CoV-2* severe acute respiratory syndrome coronavirus 2, *NHPs* nonhuman primates, *Abs* antibodies*, NAbs* neutralizing antibodies

#### Parainfluenza virus vector

##### A single IN dose vaccination of PIV vectored vaccines provide complete protection against respiratory diseases

Parainfluenza virus is a potential viral vector for its safety, genomic stability and abilities to be cultured in multiple cell lines.^44^ Multiple serotypes of PIV are involved in viral vector, including PIV1, 2, 3 and 5. In addition, B/HPIV3 is a chimeric Bovine/human PIV consisting of bovine PIV3 (BPIV3) strain Kansas in which BPIV3 HN and F glycoproteins have been replaced by those of human PIV3 strain JS.^151,152^ The BPIV3 backbone provides the host range restriction of replication in humans, which was well tolerated and immunogenic in young children.^152,153^ Till now, no evidence of enhanced pathogenicity has been confirmed in PIV vectored vaccines.^154,155^ PIVs are paediatric pathogens targeting respiratory epithelium, which made them attractive for developing vaccines that induce mucosal immune responses.^156–158^

A replication-defective COVID-19 vaccine has been developed based on human parainfluenza virus type 2 (hPIV2) vector BC-PIV, which expressed the full-length prefusion-stabilized S protein of SARS-CoV-2, termed BC-PIV/S-2PM.^159,160^ Massive S proteins were incorporated on the viral surface. A single IN dose vaccination with BC-PIV/S-2PM induced high levels of S-specific IgG and mucosal IgA antibodies in mice and protected hamsters against SARS-CoV-2 infection. Booster vaccinations were needed to confer complete protection on hamsters. Several replication-competent PIV vectored COVID-19 vaccines were also developed. CVXGA1 is a recombinant PIV5-vectored vaccine expressing S protein from SARS-CoV-2 WA1.^161^ Native configuration of the S protein was generated to maximize protective immune responses.^162,163^ A single IN dose of CVXGA1 induced viral-specific NAbs and provided 100% protection in K18-hACE2 mice and blocked contact transmission to cohoused naive ferrets. When CVXGA1 was administered as a booster following two doses of a COVID-19 mRNA vaccine, PIV5-vectored vaccines generate higher levels of cross-reactive NAbs compared to three doses of COVID-19 mRNA vaccine.^164^ These results indicate that CVXGA1 could serve as a booster vaccine against emerging variants. CVXGA1 is currently under Phase I clinical trial in the United States (NCT04954287). B/HPIV3 based COVID-19 was also constructed by expressing the native or prefusion-stabilized S protein (S-2P).^39^ Prefusion stabilization increased the expression of S proteins by B/HPIV3 in vitro. In hamsters, a single IN dose of B/HPIV3/S-2P induced 12-fold higher NAbs titers and significant higher SARS-CoV-2-specific IgA and IgG compared to B/HPIV3/S. Post SARS-CoV-2 challenge, B/HPIV3/S-2P provided better protection than B/HPIV3/S. Further, optimized version of B/HPIV3/S-2P, which involves another 4 proline mutations to consolidate the prefusion-stabilized S protein (B/HPIV3/S-6P) was evaluated in rhesus macaques.^165^ A single IN/intratracheal(IT) dose of B/HPIV3/S-6P induced strong S-specific airway mucosal IgA, IgG responses as well as high levels of peripheral S-specific antibodies, which efficiently neutralized SARS-CoV-2 VOCs, but the ability to neutralize Omicron sub-lineages was weakened. Furthermore, B/HPIV3/S-6P induced robust systemic and pulmonary S-specific CD4^+^ and CD8^+^ T cell responses, including tissue-resident memory cells in the lungs. B/HPIV3/S-6P vaccination effectively inhibited and eliminated viral proliferation in the upper and lower respiratory tract of immunized macaques. Natural attenuated human parainfluenza virus type 3 (HPIV3) vector-based COVID-19 vaccine was also proved to be effective^166,167^ In a same manner, PIV5 or B/HPIV3 vectored SARS-CoV-1 and MERS-CoV vaccines were immunogenic by a single IN dose of administration in preclinical.^159,168–170^

Human respiratory syncytial virus (RSV) is the leading viral agent of severe acute respiratory infections in infants and young children worldwide.^171^ Thus far, there is no licensed RSV vaccine. PIV-based RSV vaccines were constructed by expressing RSV-F protein from an additional transcription unit.^40,172–178^ In recombinant B/HPIV3, F protein of RSV was engineered for prefusion conformation, of which TM and CT domains were replaced of HPIV3 F to increase incorporation in vector virion.^179^ Booster with rB/HPIV3-RSV-pre-F resulted in significantly higher RSV NAbs than booster with live attenuated RSV vaccine in both hamsters and African green monkeys. PIV-based RSV vaccine provided a greater antigenic load of RSV F and increased immunogenicity compared to attenuated RSV. However, additional attenuation might make the construct over-attenuated in humans such that immunogenicity might be suboptimal.^175,179^ For these reasons, rHPIV3 JS was developed as a new generation vector to be available when rB/HPIV3-RSV-F was over-attenuated. Encouragingly, bivalent HPIV3/HRSV vaccine candidate was well tolerated in children >2 months of age, and optimized versions are in further clinical development as pediatric vaccines.^153,159,172,175^ Two RSV vaccines were constructed based on PIV5 expressing glycoproteins F (PIV5/F) and G (PIV5/G), respectively.^180–182^ PIV5/F was more immunogenic and provided better protection than PIV5/G in animal models. PIV5/F enhanced NAb responses in RSV-post exposed African green monkeys. These studies indicate that PIV5/F is a promising single-dose IN vaccine for RSV‐naive and RSV‐exposed individuals. In addition, PIV5‐based RSV vaccines could be administered subcutaneously, which provides a favorable route of vaccination for infants who may suffer from nasal congestion due to IN inoculation.

Based on the PIV platform, several IFV vaccine candidates were constructed by incorporating HA or NP of IAV H5N1 into recombinant PIV virions.^170,183–185^ A single IN dose inoculation of recombinant virus bearing HA of IAV induced sterile immunity and protected animals from homologus challenge of IFV. Compared with HA, NP of IAV seemed to be more conserved, but it was less immunogenic. This issue could be addressed by selection of appropriate locations for foreign gene delivery within the PIV genome. After that, a single IN inoculation of PIV vectored vaccine bearing NA of IFV provided broad protection against IFV. These results suggested that NP could be further investigated as a broad-spectrum antigen for IFV.

##### PIV vectored EBOV vaccines in development

Based on the HPIV3 vector, two EBOV vaccine candidates were constructed by inserting the GP gene alone or together with the NP protein gene of EBOV into the genome of HPIV3. After a single IN inoculation of the above vaccine candidates, guinea pigs were 100% protected from EBOV challenge in both vaccine groups.^186^ In rhesus monkeys, a single dose immunization with any construct expressing GP was moderately immunogenic against EBOV and protected 88% of animals against severe hemorrhagic fever and death caused by EBOV. Two doses vaccination were highly immunogenic, and all of the animals survived the challenge and were free of signs of disease and detectable challenge virus. The immune responses of PIV-based EBOV vaccines were equivalent to the AdV vector vaccine, but lower than that of the VSV vectored vaccine. Virus-specific binding antibody titer was directly related to protective efficacy. The incorporation of NP protein contribute little to the protective efficacy.^187^ Preexisting anti-vector immunity could affect replication of HPIV3, but had limited effect on the antigen expression and immunogenicity. The antibody titer against GP protein was only slightly lower in the group with pre-existing HPIV3 antibody than their counterparts. After the second immunization, antibody titers reached the equivalent level between two groups.^188,189^

Bukreyev et al. tried to remove HN and F protein from HPIV3 and replace its function with GP protein from EBOV. They successfully packaged the HPIV3 vectored EBOV vaccine without HN and F protein. The vaccine retained immunogenicity and completely protected guinea pigs against the lethal challenge of EBOV. Most importantly, the vaccine escaped pre-existing HPIV3 immunity.^36^ Deletion of HN and F protein resulted in a higher expression levels of Ebola GP protein. Meanwhile, the attenuation of the viral vector was also accomplished.

Equally, an attenuated recombinant human parainfluenza virus type 1 (rHPIV1) expressing the membrane-anchored form of EBOV GP was reported as an IN-delivered EBOV vaccine.^190^ GP was codon-optimized and expressed either as a full-length protein or as an engineered chimeric form in which its TM and CT domains (TMCT) were replaced by those of HPIV1 F protein to enhance packaging into the vector particle and immunogenicity. The GP gene was inserted either preceding the N gene (pre-N) or between the N and P genes (N-P) of rHPIV1 bearing a stabilized attenuating mutation in the P/C gene (CΔ170). These constructs grew to high titers and stably expressed EBOC GP. In addition, recombinant viruses were attenuated, which replicated at low titers over several days, in the respiratory tract of African green monkeys. Two doses of candidates expressing GP from the pre-N position elicited higher NAbs than N-P viruses, and unmodified GP induced much higher levels of NAbs than its TMCT counterpart. The unmodified EBOV GP was packaged into the HPIV1 particle, and the TMCT modification did not increase packaging or immunogenicity, but rather reduced the stability of GP expression during in vivo replication. This study indicated that TMCT replacement did not always enhance ectopic protein incorporation and the immunogenicity of the vaccine, which was determined by attribute of specific pathogen. Representative PIV vector-based vaccines for human disease were summarized in Table 4.

**Table 4:** Vaccines based on parainfluenza virus vector

Pathogens	Design strategy	Stage	Results	Advantages	Overall concerns	Reference
SARS-CoV	B/HPIV3-S	Hamsters and NHPs	Protected from disease and detectable viral replication	Single dose	\	^159,168^
SARS-CoV-2	hPIV2-prS	Mice and hamsters	Protected from disease and detectable viral replication	Single dose; massive spike proteins incorporation; mucosal immunity	Two doses needed to complete protection in nasal turbinates	^160^
	PIV5-S(CVXGA1)	Mice and ferrets	100% protection or protected from the contact transmission	Single dose; broad spectrum; well-maintained NAbs; mucosal immunity; tissue-resident memory cells	\	^161,164^
	B/HPIV3-prS	Hamsters and NHPs	Protected from disease and detectable viral replication	Single dose; broad spectrum neutralizing; mucosal immunity; spike proteins incorporation	\	^39,165^
	HPIV3-S/S1/RBD	Hamsters	Protected from disease and detectable viral replication	Single dose; HPIV3-S was selected as the best construct in terms of immune response; safe	\	^166,167^
MERS-CoV	PIV5-S	Mice	100% protection	Single dose	\	^169,170^
RSV	B/HPIV3-F	Phase I	Immunogenicity and well-tolerated	Single dose; safe; applicable to infants and children; bivalent	\	^153,159,172,175,179,535^
	PIV5-F/G	Mice, cotton rats and NHPs	Protected from disease and detectable viral replication	Single dose; PIV5-F was selected; applicable for RSV-exposed persons	Pre‐fusion RSV‐F do not enhance immune response	^180–182^
IFV	PIV5-HA/NP	Mice	67–100% protection	Single dose; broad spectrum; optimized insertion site was selected;	Incomplete protection of NA as immunogen	^170,183–185^
Ebola virus	HPIV3-GP/GP + NP	Guinea pigs and NHPs	100% protection	Single dose; limited effect about pre-existing immunity	Immune response lower than VSV vectored vaccine	^.^^137,186–189^
	hPIV2-GP	Mice	NAbs	Low pathogenicity and recurrent infections of parental hPIV2	\	^27,536^
Rabies virus	PIV5- G	Mice	50–100% protection	Single dose; protective immune responses via IN, IM, and OR immunization	\	^339^

*SARS-CoV* severe acute respiratory syndrome coronavirus, *SARS-CoV-2* severe acute respiratory syndrome coronavirus 2, *MERS-CoV* Middle East respiratory syndrome coronavirus, *RSV* respiratory syncytial virus, *IFV* influenza virus, *NHPs* nonhuman primates, *NAbs* neutralizing antibodies, *IM* intramuscular, *IN* intranasal, *OR* oral, *VSV* vesicular stomatitis virus

#### Measles virus vector

Live attenuated measles virus (MeV) vaccine was one of the most effective and safe human vaccines in clinical.^191^ Accordingly, the manufacturing industry of MeV vaccines is mature enough. Given the significant success of the MeV vaccine, this virus was considered a backbone for viral vectored vaccines against other diseases. Among them, MeV strain Schwarz and Moraten were frequently applied backbones. Notable progress has been made in MeV-based vaccines.

##### MeV-based vaccines expressing distinct forms of antigens provide protection against respiratory diseases

Homologous prime-boost immunization with replication-competent rMeVs expressing the S glycoprotein of MERS-CoV, either in its full-length, truncated or soluble variant, induced robust levels of both rMeV- and MERS-CoV NAbs and T cells in MeV susceptible mice.^192^ Post challenge with MERS-CoV, viral loads in the lungs of vaccinated mice were significantly reduced, coinciding with reduced pathological alterations in the lung, suggesting that rMeV-MERS vaccines confer full protection against MERS-CoV infection. The expression of the soluble version of S by MeV did not enhance NAb titers and slightly impaired replication in contrast to MeV expressing full-length MERS-S. These results indicated that the soluble structure of the S protein hampered the assembly of the recombinant virus. In a same manner, rMeV expressing codon-optimized S glycoprotein (S) SARS-CoV is immunogenic in mice.^193^

Several attempts have been made to develop MeV-based COVID-19 vaccines. These preclinical candidates were constructed by harboring membrane-anchored wild-type S protein, the pre-fusion stabilized S protein (S-2P) or secreted form of S-2P with a self-trimerizing “foldon” domain. Both of them were claimed to be effective in animal models.^194–196^ Besides, the new version was also designed to encode prefusion-stabilized, trimerized SARS-CoV-2 S glycoproteins displayed on a dodecahedral miniferritin scaffold. Surface glycoproteins of MeV were modified to bypass anti-measles antibodies. The optimized version of the MeV-based COVID-19 vaccine induced a high titer of NAbs in mice. These antigen-engineering strategies may also be applicable to measles-based vaccines for other emerging beta coronaviruses.^197^ Unfortunately, immunogenicity was insufficient after a single IM dose of MeV-based COVID-19 vaccine expressing a pre-fusion stabilized SARS-CoV-2 S protein (V591) in Phase I/II clinical trials, especially in measles-immunized individuals.^198,199^ Currently the relationship between low immunogenicity and anti-vector immunity is not clear. Most importantly, IP inoculations were conducted in animal models, while IM inoculations were applied in clinical trials, which may help explain the conflicting results between preclinical and clinical trials.

Apart from the above strategy that involved another transcription unit to co-express the foreign antigen, a chimeric version of MeV was also constructed, in which the CT and TM domains of MeV F and H was maintained, while ectodomains of MeV F and H were substituted by RSV F and G, correspondingly.^200^ The chimeric MeV/RSV induced NAbs against RSV in cotton rats and significantly reduced viral loads after challenge. The ectodomain replacement strategy may be similarly practicable for other paramyxoviruses, done under critical monitor since the change of entry receptor tropism.

##### MeV-based vaccines for vector-borne diseases

West Nile virus (WNV) is an arthropod-borne flavivirus that causes numerous cases of human encephalitis. MeV-based vaccine candidate (MeVSchw-sE) was constructed by expressing envelope glycoprotein from WNV. An IP dose inoculation with MeVSchw-sE induced both high levels of specific anti-WNV NAbs and protection from lethal challenge of WNV in mice and squirrel monkeys.^201,202^

Chikungunya virus (CHIKV) is a mosquito-borne alphavirus that causes severe polyarthralgia. rMeV expressing CHIKV capsid and envelope structural proteins resulted in the formulation of virus-like particles (MeV-CHIKV). MeV-CHIKV elicited broad spectrum and high titers of CHIKV antibodies as well as cellular immune responses. All mice survived the lethal challenge of CHIKV post a single IP dose of immunization.^203^ Passive transfer of immune sera conferred protection to naïve mice, highlighting the essential role of humoral immune response in protecting CHIKV. The final preclinical evaluation of MeV-CHIKV was performed in cynomolgus macaques. Homologous prime-boost vaccination with MeV-CHIKV protected macaques from abnormal clinical signs, viremia, blood cell indicators, cytokine changes upon challenge with CHIKV.^204^

This Schwarz strain-based rMeV encoding CHIKV VLPs has undergone Phase I/II clinical trials. MeV-CHIKV was well tolerated and immunogenic despite pre-existing anti-MeV immunity, with immunity persisted up to 6 months.^205,206^ Two doses are required for 100% seroconversion rates. Moreover, the vaccine boost at 6 months appeared to increase NAb titres to a greater extent. MeV-based Lassa fever vaccines were constructed by expressing GPC, GPC + NP or GPC + Z proteins of LASV, respectively. In cynomolgus monkeys, MeV-GPC + NP was determined as the optimal schedule after a single subcutaneous (SC) dose of vaccination in terms of immune response and post-challenge protective efficacy.^207^ Further evaluation confirmed that a single SC dose of MeV-GPC + NP protected cynomolgus monkeys from both homologous (Josiah, lineage IV) and heterologous (lineage II and lineage VII) strains of LASV. One year post a single dose of MeV-GPC + NP vaccination, 100% of monkeys were protected from homologous lethal challenge. These studies suggested that MeV-GPC + NP confer long-term and broad-spectrum protection against LASV.^208^ Currently, the Phase I clinical trial of MeV-GPC + NP is ongoing (NCT04055454).

Given the ideal results of a VSV-based vaccine co-expressing prM and E protein of ZIKV, a recombinant MeV encoding ZIKV prM and soluble E proteins (MV-Zika-sE) was constructed. Mice were inoculated with two doses of MV-Zika-sE via IP injection. MV-Zika-sE vaccinated mice were protected from weight loss and plasma viremia.^209^ There has also been attempts to screen a panel of MeV-based vaccine constructs expressing ZIKV-E, NS1, or both. Although MeV-E2 provided a 100% survival rate in mice, complete viral clearance was not achieved. NS1 was required to provide full protection. Representative MeV vector-based vaccines for human disease were summarized in Table 5.

**Table 5:** Vaccines based on measles virus vector

Pathogens	Design strategy	Stage	Results	Advantages	Overall concerns	Reference
SARS-CoV	rMeV-coS	Mice	Immunogenic	\	\	^193^
SARS-CoV-2	MeV-S/S-2P/secreted S-2P/self-trimerizing S displayed on miniferritin	Mice and hamsters	Immunogenic and protected animals from disease and detectable viral replication	Safe, less influenced by anti-vector immunity	Lack of convenient animal model; contradictory results in preclinical and clinical trials	^194–197^
		Phase I/II	Well-tolerated but less well immunogenic	Safe	Reconsidering of delivery route or design strategy	^198,199^
MERS-CoV	MeV-S/S(truncated)/S(soluble)	Mice	Immunogenic and protected animals from disease and detectable viral replication	Vaccinated animals were fully protected	Soluble version of S impaired replication of rMeV	^192^
RSV	MeV/RSV	Cotton rats	Immunogenicity and reduce virus load in respiratory tract	Chimeric version of MeV whose ectodomains of F and H were substituted by the RSV F and G, while CT and TM domain were maintained	Changing of cell tropism should be monitored	^200^
CHIKV	MeV-CHIKV capsid+envelope	Mice and cynomolgus macaques	Immunogenic and protected animals from disease	Formulation of virus-like particles; broad-spectrum NAbs; highlight the role of humoral immune response in protection	/	^203,204^
		Phase I/II	Well-tolerated and immunogenic	Less influenced by anti-measles antibodies; immune response persisted up to 6 months	/	^205,206^
WNV	MeV-envelope	Mice and squirrel monkeys	Immunogenic and protected animals from lethal challenges	Single dose regime	/	^201,202^
LASV	MeV-GPC/GPC + NP/GPC + Z	Cynomolgus monkeys	Immunogenic and protected animals from disease	MeV-GPC + NP was determined as the optimal schedule; broad spectrum and long-term protection for 1 year	MeV-based vaccine expressing VLPs doesn’t always work	^207,208^
ZIKV	MeV-prM+E	Mice	Immunogenic and protected animals from weight loss and viremia	/	NS1 is needed for fully protection	^209,537^

*SARS-CoV* severe acute respiratory syndrome coronavirus, *SARS-CoV-2* severe acute respiratory syndrome coronavirus 2, *MERS-CoV* Middle East respiratory syndrome coronavirus, *RSV* respiratory syncytial virus, *CHIKV* Chikungunya virus, *WNV* West Nile virus, *LASV* lassa fever virus, *ZIKV* zika virus, *S-2P* pre-fusion stabilized spike protein with two proline mutations. nonhuman primates, *MeV* measles virus, *S* spike, *E* envelope protein, *GPC* glycoprotein precursor, *NP* Nucleoprotein, *Z* zinc finger protein, *F* fusion protein, *H* hemagglutinin glycoprotein, *CT* cytosolic tail, *TM* Transmembrane, *NAbs* neutralizing antibodies, *NS1* non-structure protein 1

#### Newcastle disease virus vector

Newcastle disease virus (NDV) is another highly contagious paramyxovirus that could cause varying disease severity in avians but behaves strict host restrictions.^210^ In mammals, NDV triggered interferon responses, which restricted the replication of NDV and simultaneously posed an adjuvant effect on adaptive immunity.^211,212^ Low‐virulence NDV strains, such as LaSota and B1, are widely used as live attenuated vaccines for lethal NDVs and engineered for veterinary and human vaccines.

NDV-based SARS-CoV vaccine was developed by expressing the S protein of SARS-CoV from an added transcriptional unit.^213^ After two IN doses vaccination, African green monkeys developed high titers of NAbs against SARS-CoV. Post a high-dose challenge of SARS-CoV, viral titer in lung tissue was significantly reduced compared to control animals.

NDV vectored COVID-19 vaccine has been constructed and evaluated in preclinical and clinical. Previous antigen-engineering strategy re-occurred in NDV-based COVID-19 vaccines, including stabilizing S protein by the introduction of 6 prolines and adding TM and CT domains of NDV fusion protein to enhance the expression of S protein on the surface of the viral particles.^165,179^ Representatively, rNDV‐S was constructed by expressing S protein of SARS-CoV-2 based on NDV vector. In mice, rNDV‐S induced both humoral and cellular immunity through IM immunization, while no NAbs were detected despite a higher S‐specific T‐cell response induced by IN injection.^214,215^ Similarly, rNDV‐S was less immunogenicity through solely IN inoculation compared to IM inoculation in pigs despite the combination of the two delivery routes inducing strong NAbs. Interestingly, live rNDV‐S via IN inoculation induced antibody response and protective efficacy comparable to IM inoculation in hamsters.^216^ These proof-of-concept studies illustrated the animal model-dependent manner of the rNDV‐S vaccine, emphasizing the need for clarification of animal models that accurately reflect the status in human beings post vaccination.^217^

Inactivated rNDV‐S was evaluated in Phase I clinical trials, which proved safe and immunogenic.^218,219^ Indeed, this vaccine candidate could be inexpensive and scalable in manufacturing. However, inactivated NDV-based vaccines seem to be less attractive than novel protein vaccines for COVID-19.^220–222^ Live rNDV‐S was also evaluated in prime-boost regimens via IM, IN, or IN followed by IM routes in Phase I clinical trial. Live rNDV‐S was safe and well tolerated. IM inoculation and IN followed by IM administration were proved to be immunogenic.^223^ Superficially, preclinical evaluation of rNDV‐S in pigs seems better reflect clinical outcomes in humans. However, complicated issues should be addressed as inequality exists in these IN delivery routes. For IN inoculation, humans and pigs were given by nasal sprayer device, while hamsters were given under anesthesia, which enable the deeply distributed of rNDV‐S and represented more likely those in aerosol inhalation vaccines.^216^ Therefore, clinical trial should be designed and handled carefully, in case that delicate divisions in the delivery route exist.

Bivalent rNDV vaccines have been developed by targeting both NDV and highly pathogenic avian influenza (HPAI) by expressing chimeric HA from IFV. These vaccine candidates could provide cross-protection between different IFV lineages.^224,225^ Similar in NDV-based COVID-19 vaccine, inactivated rNDV was more immunogenic through IM inoculation than that of IN inoculation.^225,226^ This could be explained by the mucosal tropism of live NDV, while inactivated NDV display more antigen proteins and benefit from adjuvant effect. Currently NDV-based IFV vaccine is used as veterinary vaccines in Mexico.

Given that NDV is a potent inducer of interferon production and dendritic cell maturation, a recombinant NDV expressing RSV fusion glycoprotein was administered to BALB/c mice. A single IN dose of vaccination protected animals from the RSV challenge.^227^ Further evaluation of cotton rats showed that vaccination also protected them from RSV challenge and induced long-lived NAbs up to 6 months.^228^

To compare with the PIV3-based EBOV vaccine, the same team developed an NDV-based EBOV vaccine expressing EBOV GP, termed NDV/GP. Following one IN plus IT dose inoculation with NDV/GP, EBOV-specific binding antibodies and NAbs were undetectable or low compared to those induced by HPIV3/GP in rhesus monkeys. Boosting vaccination led to a substantial increase in serum IgG ELISA titers, yet remained lower than those induced by a second dose of HPIV3/GP. In contrast, secretory IgA titers in the respiratory tract and NAbs were equal to those induced after the second dose of HPIV3/GP. These results suggested that NDV-based EBOV vaccine was equivalent to or slightly less immunogenic than PIV3-based EBOV vaccine, particularly in the single-dose regimen.^229^ To overcome the anti-vector immunity of Ad5, rNDV was generated by expressing the GP protein of the EBOV and was combined with AdV-5-MakGP as a heterologous prime-boost strategy.^230^ This strategy exhibited more-potent EBOV GP-specific antibodies and cellular immune responses than those received the same vaccine twice in mice. These results suggest that the AdV-5 prime-NDV boost regimen is more effective in stimulating EBOV-specific immunity than the homologous regimen. Representative NDV vector-based vaccines for human disease were summarized in Table 6.

**Table 6:** Vaccines based on Newcastle disease virus vector

Pathogens	Design strategy	Stage	Results	Advantages	Overall concerns	Reference
SARS-CoV	rNDV-S	Mice	Immunogenic and reduced virus load	\	\	^213^
SARS-CoV-2	rNDV-S/S-6P/	Mice, hamsters and pigs	Immunogenic and protected animals from disease and detectable viral replication	Pre-fusion stabilized; both live and inactivated forms are available; IN plus IM inoculation	Poor immunogenicity through IN inoculation	^165,179,214,215^
		Phase I	Well-tolerated and immunogenic	Both live and inactivated rNDV are safe and immunogenic; IN prime-IM boost strategy		^218,219,223^
IFV	rNDV-HA	Avian	Immunogenic and protected animals from lethal challenge	Bivalent; cross-protection	\	^224,225,229^
RSV	rNDV-F	Mice and cotton rats	Immunogenicity and protected animals from challenge	Single dose; long-lasting NAbs response (6 months)	\	^227,228^
EBOV	rNDV-GP	Cynomolgus macaques	Immunogenic	Comparable or slightly lower immunogenic than HPIV3/GP	Poor immunogenicity through a single IN dose inoculation	^229^
	rNDV+Ad5-GP		Immunogenic	Heterologous prime-boost strategy	\	^230^
RVFV	rNDV-GnGc	Mice and lambs	Immunogenic and protected animals from challenge	\	\	^538^
NIV	rNDV-F/G	Mice and pigs	Immunogenic	Co-immunization with rNDV-F and rNDV-G; long-lasting immune response	\	^539^

*SARS-CoV* severe acute respiratory syndrome coronavirus, *SARS-CoV-2* severe acute respiratory syndrome coronavirus 2, *IFV* Influenza virus, *RSV* respiratory syncytial virus, *EBOV* Ebola virus, *RVFV* rift valley virus, *NIV* Nipah virus, *rNDV* recombinant new castle disease virus, *S* spike, *S-6P* pre-fusion stabilized spike protein with 6 proline mutations, *HA* hemagglutinin glycoprotein, *F* fusion protein, *GP* glycoprotein, *GnGc* glycoprotein of rift valley virus, *NAbs* neutralizing antibodies, *NS1* non-structure protein, *IM* intramuscular, *IN* intranasal, *HPIV3/GP* human parainfluenza virus type III vector-based EBOV vaccine expressing EBOV GP

#### Influenza virus vector

##### IN-delivered IFV-based COVID-19 vaccine is potent when standing alone or as booster vaccines

The existing influenza vaccines production infrastructure is highly optimized and capable of delivering more than a billion doses per year.^231^ To combat another respiratory disease, COVID-19, multiple vaccines based on IFV vectors have been developed. scPR8-RBD-M2 was designated as a single-round replication IFV-based COVID-19 vaccine. Chimeric gene was utilized to encode 2 A peptide-based bicistronic protein cassette of the SARS-CoV-2 RBD and IFA M2. The C-terminus of the RBD was linked to the cytoplasmic domain of IFV HA to anchor the RBD to the surface of producing cells and the virus envelope. Cellular, humoral and mucosal immune responses to RBD can be produced in mice with two doses of IN immunization. Vaccination-induced antibodies represented broad-spectrum neutralizing activity against SARS-CoV-2 variants.^232^ HA protein provided by MDCK-HA cells may lead to instability of the inserted gene maintenance. To address this issue, a vaccine cocktail that contained mixed antigens/epitopes of interest could be generated to circumvent such limitations. Chaparian et al. inserted SARS-CoV-2-RBD into IFV A/Puerto Rico/8/1934 (H1N1) HA, vaccination with this combination vaccine elicited NAbs and provided protection against the lethal challenge of both SARS-CoV-2 and IAV in mice.^233,234^

More recently, a live-attenuated SARS-CoV-2 vaccine was manufactured based on a cold-adapted IFV strain without NS1, in which the RBD gene of SARS-CoV-2 was inserted by gene reassortment, termed CA4-dNS1-nCoV-RBD (dNS1-RBD).^62,235^ In preclinical studies, dNS1-RBD induced rapid, long-term, broad-spectrum protection against SARS-CoV-2 challenge in hamsters by inducing strong innate and adaptive local immune responses in the respiratory tract, despite weaker responses in the circulation, which might be attributable to innate immune response in the nasal epithelium and local cross-variant specific T-cell immune response.^235^ Lung-resident memory RBD-specific CD4^+^ and CD8^+^ T cells could be induced by vaccination, and the T-cell immune response produced in lung tissue was about 26-time stronger than that in peripheral mononuclear cells (PBMCs) in mice immunized with a single dose. Moreover, such cellular immunity is relatively unimpaired for most SARS-CoV-2 VOCs, especially for the latest Omicron variant. In addition, this vaccine also provides cross-protection against IFV H1N1 and H5N1. In Phase I/II clinical trials, dNS1-RBD was administered by IN inoculation in healthy adults.^14^ dNS1-RBD was well tolerated in adults, less than 20% of vaccine-related adverse reactions were observed, no serious adverse event was noted. In the Phase I/II trial, specific T-cell immune responses, seroconversion for RBD-specific IgG and positive conversion for RBD-specific s-IgA were observed at 44%, 10% and 12%, respectively, in vaccine recipients 1 month after the second dose. Overall, T-cell, humoral and mucosal immune responses to SARS-CoV-2 were weak in vaccine recipients. This study provided evidence of cross-contamination caused by aerosols of the IN vaccine produced during administration, which could help pave the way for the clinical development of other IN vaccines in the future. Although the probability of vaccine strain transmission through close contact with a vaccinated person is believed to be very low.^236^ This issue should be properly addressed by the assessment of viral shedding and specific immune responses in vaccinators, probability of environmental infection. Phase III clinical trials of dNS1-RBD are ongoing (ChiCTR2100051391). Notably, broad-spectrum efficacy against Omicron has been achieved. The overall protective efficacy of dNS1-RBD against hospitalizing of COVID-19 was 100%. For people without immunization history, the absolute protective effect of dNS1-RBD at 3 months after immunization was 55%. For people with immunization history, the absolute protective efficacy of nasal spray COVID-19 vaccine within 6 months after booster immunization was 82% (unpublished data). On December 2, 2022, dNS1-RBD was approved for emergency use in China.

##### IFV-vectored vaccines for other pathogens

Cold-adapted, live-attenuated influenza vaccine (CAIV; FluMist, AstraZeneca, London UK) was licensed as a safe and effective vaccine by the US Food & Drug Administration in 2003 and is approved for use in people aged 2–49 years.^237^ In a human challenge trial of FluMist, a low antibody response was not directly associated with low protective efficacy.^238^ Among 103 adults aged 18–45 years who received a single dose, the seropositive rates of haemagglutination-inhibiting antibodies for IAV/H1N1, IAV/H3N2, and IBV/Harbin were 23%, 33%, and 3%, and the response rates of IgA antibodies in nasal wash were 14%, 32%, and 18%, respectively. Encouragingly, the virus challenge results indicated that the protective effects of FluMist for A/H1N1, A/H3N2, and B/Harbin were 80%, 78%, and 100%, respectively, which were higher than those of IM vaccine candidates (60%, 67%, and 100%) that inducing higher seroresponse rates (91%, 76%, and 76%). Likewise, the PIV5/G vaccine did not produce detectable levels of NAbs in cotton rats but still provided protection against RSV challenge.^180^ The above results suggested that immune responses other than peripheral antibody responses may provide benefits of protection against these respiratory diseases.

Our group constructed an H5N1 chimeric IAV/B vaccine based on a cold-adapted (ca) IBV B/Vienna/1/99 backbone.^239^ Modified HA of H5N1 was inserted while the packaging signals of HA of IBV were retained. The recombinant virus maintained a temperature-sensitive and cold-adapted phenotype. The H5N1 vaccine was attenuated in mice. Systemic humoral and cellular immunity and local mucosal IgA were induced. Two-dose IN vaccination of the chimeric H5N1 vaccine candidate conferred full protection against the lethal challenge of IFV H5N1 in mice. In 2021, a conserved extracellular domain of IFV ion channel protein M2 (M2e) (4 × M2e) was inserted into the N terminal of A/Switzer land/9715293/ 2013 (H3N2) HA. Intranasally inoculation of this vaccine induced antibodies and T cell immune response in mice, thus achieving protection against H1N1, H3N2, H5N1, H7N9 and H9N2 viruses.^240^ Representative IFV vector-based vaccines for human disease were summarized in Table 7.

**Table 7:** Vaccines based on influenza virus vector

Pathogens	Design strategy	Stage	Results	Advantages	Overall concerns	Reference
SARS-CoV-2	HA-RBD-M2	Mice	Protected from the disease and detectable viral replication	Broad spectrum neutralizing activity; local and systematic immunity	Instability of the inserted gene maintenance	^232^
	HA-RBD	Mice	Protected from the disease and detectable viral replication	Protect against both SARS-CoV-2 and IAV	Instability of the inserted gene maintenance	^233^
	CA4-dNS1-nCoV-RBD (dNS1-RBD)	Hamsters	Protected from the disease and detectable viral replication	Rapid, long term, and broad-spectrum protection; innate and adaptive local immune responses	Weaker responses in circulation	^235^
		Phase I/II	Well tolerated	<20% vaccine-related adverse reactions	T-cell, humoral and mucosal immune responses against SARS-CoV-2 were weak in recipients; cross-contamination	^14^
		Phase III	100% protection against hospitalization	55% and 82% protection for people without/with immunization history	\	Unpublished
IFV	Live attenuated (FluMist)	Phase III	78–100% protection	Low level of NAbs but provide effectively protection	\	^237,238^
	Chimeric IBV-HA(IAV)	Mice	100% protection	Cold adaption; attenuated; systemic and local immune response	Poor binding IgGs	^239^
RSV	HA-F243-294	Mice	Protected from the disease and detectable viral replication	Single dose; no ADE effect	Poor NAbs	^540^
WNV	NA-DIII	Mice	Humoral and cellular immunity	\	\	^541^

*SARS-CoV-2* severe acute respiratory syndrome coronavirus 2, *IFV* influenza virus, *RSV* respiratory syncytial virus, *WNV* West Nile virus, *IBV* influenza B virus, *IAV* influenza A virus, *NAbs* neutralizing antibodies, *ADE* antibody-dependent enhancement

#### Adenovirus vector

##### Homologus or heterologous primer-boost of AdV based vaccines provide protection against viral haemorrhagic fever

Adenovirus type 5 (Ad5) is the most frequently applied adenovirus vector, which is well established and easily accessible. In preclinical trials, cynomolgus macaques were boosted with the replication-defective Ad5-vectored vaccine candidate Ad5-EBOV encoding EBOV GP after initial immunization with the DNA vaccine. These animals generated vigorous cellular and humoral immunity and received full cross-protection.^241,242^ Passive transfer of polyclonal antibodies from vaccinated animals to naive macaques failed to confer protection against the lethal challenge of EBOV, while depletion of CD8^+^ cells in vivo abrogated protection for NHPs.^243^ These results indicated that CD8^+^ T cells play a major role in rAd5-EBOV induced immune protection against EBOV infection. In Phase I clinical trial, Ad5-EBOV was safe and immunogenic.^244^ However, humoral responses were impacted by pre-existing anti-vector immunity. Likewise, a single IM dose of Ad5-MakGP, a recombinant Ad5 expressing the GP of EBOV Makona strain, provided sterile immunity and 100% protection for NHPs.^245^ In Phase I clinical trial, Ad5-MakGP showed good safety and immunogenicity. Dose-dependent magnitude of immune response was observed. Both the EBOV-specific antibody response and T-cell response were blunted by the presence of anti-vector immunity, particularly in the low-dose group.^246,247^ One homologous booster immunization with Ad5-MakGP at month 6 after primary immunization stimulated a stronger humoral immune response. One year after booster immunization, a 100% positive rate of GP antibody remained to be detected.^248^ According to clinical outcomes in Phase II clinical trials of Ad5-MakGP in Sierra Leone, when the vaccination dose was increased to 8 × 10^10^ viral particles, adverse reactions to vaccination were acceptable and the incidence rate was even lower than in Phase I clinical trial.^249^ Whereas, the duration of EBOV-specific antibodies in African participants was shorter than in Chinese participants, also seen in clinical trials of rVSV-ZEBOV in Africa and Europe.^95^ In Phase I/II clinical trials, the rVSV-ZEBOV + Ad5-EBOV prime-boost regime induced a robust immune response.^250^ Heterologous prime-boosting strategy could quickly awaken immune memory and induce a stronger immune response, simultaneously alleviating the influence of anti-vector immunity. Russia approved the registration of this vaccine in December 2015.

Adenovirus type 26 (Ad26) is another promising vaccine vector with lower seroprevalence than Ad5. Ad26-based EBOV vaccine was also constructed. A single IM dose vaccination of Ad26-ZEBOV vaccine candidate expressing ZEBOV GP conferred partial protection in NHPs. Subsequently boosted immunization with Adenovirus type 35 (Ad35)-ZEBOV significantly increased humoral and cellular response and conferred complete protection.^251^

Regarding high-quality and magnitude of immune responses induced by recombinant Chimpanzee Adenoviruses (ChAdVs), they were equally applied as viral vectors.^252–254^ ChAdVs-based vaccine aroused comparable humoral and cellular immune responses to human AdV vectors.^255–257^ Chimpanzee adenoviruses type 3 (ChAd3)-vectored bivalent vaccine (cAd3-EBO) encoding GPs of EBOV and SUDV induced superior humoral and cellular responses and conferred uniform protection against EBOV challenge for macaques compared to chimpanzee adenoviruses type 63 (ChAd63) and (MVA) vectored vaccines.^258^ Boosted cAd3-EBO with MVA-vectored vaccine generated long-lasting protection against lethal challenge in NHPs.^258^ Acute protection was strongly associated with antibody responses, while long-term protection required the generation of both effector and memory CD8^+^ T-cell response and cytokines. In Phase I clinical trial, cAd3-EBO was safe and induced dose-dependent immune responses.^257^ ChAd3-based monovalent vaccine (ChAd3-EBO-Z) encoding the GP of ZEBOV was also constructed. In a Phase I clinical trial, antibodies induced by ChAd3-EBO-Z were slightly lower than those induced by rVSV-ZEBOV.^259^ When ChAd3-EBO-Z was boosted with MVA-EBO-Z, virus-specific antibodies and CD8^+^ T cells were increased by 12 and 5 times, respectively. Virus-specific antibody responses in participants primed with ChAd3-EBO-Z remained positive 6 months post immunization but were significantly lower than those who received MVA-EBO-Z booster.^259^ Other Phase I trials validated the safety and immunogenicity of ChAd3-EBO-Z.^260,261^ In addition, prime-boost strategy involving ChAd3-EBO-Z and MVA-BN-Filo (MVA-vectored vaccine candidate expressing ZEBOV GP, SUDV GP and MARV-Musoke GP) conferred long-lasting protection.^260^ Immune responses were largely maintained through 12 months.^262,263^

A DNA prime-Ad5 boost strategy was also conducted on MARV. Based on DNA or Ad5 platform, vaccine candidates DNA-MARV-GP and rAd5-MARV-GP were constructed by expressing EBOV GP. In NHPs, the protective efficacy of heterologous DNA-MARV-GP/rAd5-MARV-GP prime-boost strategy, single-dose rAd5-MARV-GP regimen, and DNA-MARV-GP homologous prime-boost strategy were compared.^264^ All three programs prevented the lethal challenge of EBOV in NHPs. A single-dose inoculation of rAd5-MARV-GP induced humoral and cellular responses comparable to those induced by two doses of DNA vaccine. Vaccine regimens containing rAd5-MARV-GP, either alone or as a booster, exhibited CD8^+^ T-cell dominant cellular responses. The dominance of the CD8^+^ T-cell subset was positively associated with a low frequency of clinical signs, suggesting that both the magnitude and functional phenotype of CD8^+^ T cells determined the vaccine efficacy against MARV infection.^264^

Maruyama et al. developed an Ad5-vectored vaccine candidate expressing LASV-GPC, termed Ad5-LASV. Two IM doses of Ad5-LASV provided complete protection for guinea pigs. All vaccinated animals produced anti-GP antibodies, while only 37.5% produced NAbs. No detectable viruses were observed in vaccinated guinea pigs post lethal LASV challenge.^265^ Zivcec et al. constructed an Ad5-based vaccine candidate Ad-CCHFV-N which the nucleocapsid protein of CCHFV.^266^ Ad-CCHFV-N induced anti-N humoral immune response. Single dose vaccination with Ad-CCHFV-N provided 30% protection for IFNAR^−/−^ mice against the lethal CCHFV challenge, while the prime-boost regimen increased protection efficacy to 78%. This study demonstrated the feasibility of genetically conserved N protein as a protective antigen against CCHFV.

ChAdOx1 is a replication-deficient chimpanzee adenovirus vector that is phylogenetically classified as Human adenovirus E.^267^ Warimwe et al. constructed a replication-deficient chimpanzee adenovirus vectored RVF vaccine termed ChAdOx1-GnGc, which encodes Gn and Gc of RVFV.^268^ ChAdOx1-GnGc induced potent RVFV-specific NAbs and CD8^+^ T-cell response in mice.^269^ A single dose of each vaccine candidate protected mice from lethal RVFV challenge. Meanwhile, two commercially available adjuvants, Matrix-M™ and AddaVax™, were demonstrated to significantly enhance the RVFV-specific neutralizing response induced by ChAdOx1-GnGc. A single dose vaccination of ChAdOx1-GnGc elicited robust NAb comparable to the licensed livestock vaccine Smithburn and conferred full protection against challenge in the most susceptible natural target species sheep, goats and cattle.^270^

##### Remarkable progresses achieved in AdV-based COVID-19 vaccines

During the pandemic of COVID-19, vaccine candidate Ad5-nCoV was designed to deliver the S protein of SARS-CoV-2. In Phase I/II clinical trial, a single IM dose vaccination of Ad5-nCoV was tolerable and immunogenic in healthy adults, binding antibodies, NAbs and SARS-CoV-2 specific T-cell responses were induced. However, preexisting anti-vector immunity compromised seroconversion of SARS-CoV-2 NAbs and reduced post-vaccination T-cell responses.^271,272^ The results of the Phase III clinical trial suggested that 14 or 28 days after a single IM dose injection of the vaccine, the overall protective efficacy was 68.83% and 65.28%, respectively. The protective efficacy against severe illness 14 or 28 dpi was 95.47% and 90.07%, respectively.^11^ On February 5, 2021, the conditional listing application for Ad5-nCoV was approved.

Harvard Medical School constructed an Ad26-vectored COVID-19 vaccine termed Ad26.COV2.S, which contained the wild-type SARS-CoV-2 leader sequence, the full-length membrane-bound S with a mutation in the furin cleavage site and two proline stabilizing mutations.^273^ A single IM dose of Ad26.COV2.S induced robust NAbs and provided complete or near-complete protection in bronchoalveolar lavage and nasal swabs following the SARS-CoV-2 challenge in NHPs. Russia developed and tested the single-dose rAd26 vector-based COVID-19 vaccine (Sputnik Light) in a Phase I clinical trial, which showed a good safety profile and induced a strong humoral and cellular immune responses in both seronegative and seropositive participants.^274^ According to the efficacy and safety analysis of single-dose Ad26.COV2.S in Phase III clinical trial, Ad26.COV2.S provided 52.9% protection against moderate to severe critical COVID-19, the protection sustained for 6 months.^12^

The University of Oxford developed a ChAdOx1-vectored COVID-19 vaccine encoding the codon-optimized full-length S gene, termed ChAdOx1-S.^275^ In rhesus macaques, ChAdOx1-S induced certain levels of immune response, but the protective efficacy was not ideal.^276^ The prime-boost regimen significantly enhanced antibody and T-cell responses in pigs but not mice compared to the single-dose group. In Phase I/II and II/III clinical trials, ChAdOx1 nCoV-19 was tolerated, humoral and cellular immune responses were observed in most volunteers, comparable T-cell responses were induced in HIV-infected individuals.^277–279^ Antibody and protective efficacy lasted at least 3 months. The overall efficacy of two-dose vaccinations was 70.4%.^13^ However, the Phase III clinical trial was once called off due to a case report of transverse myelitis. What’s more, vaccination associated-thrombus is possible.^280^ These safety issues of ChAdOx1 require further evaluation.

Heterologous prime-boost strategy is an effective countermeasure to alleviate anti-vector immunity. Russia developed rAd26 and rAd5 vector-based heterologous prime-boost COVID-19 vaccines, termed Sputnik V.^281^ Compared to the single-dose strategy, the heterologous rAd26 and rAd5 vector-based COVID-19 vaccine induced significantly stronger humoral and cellular immune responses in participants. In Phase I/II studies, Sputnik V induced pronounced humoral responses in all participants, with a 100% seroconversion. In the Phase III trial involved almost 20,000 subjects, a 91.6% protective efficacy was reported.^15^ Representative AdV vector-based vaccines for human disease were summarized in Table 8.

**Table 8:** Vaccine candidates based on adenovirus vectors

Pathogens	Design strategy	Stage	Results	Advantages	Overall concerns	Reference
Ebola virus	Ad5-(Zaire + SUDV) GP	Phase I	Safe and immunogenic	Bivalent	Anti-vector immunity	^244^
	Ad5-Zaire (Makona) GP	Phase II	Safe and immunogenic	Sterile immunity	Anti-vector immunity	^245–247,249^
	rVSV-GP + Ad5-GP	Phase II	Safe and immunogenic	Alleviated anti-vector immunity	\	^250^
	Ad26-ZaireGP + MVA-BN-Filo	Phase I	Safe and immunogenic	Antibodies persisted to 1 year	\	^285,286^
	ChAd3-(Zaire + SUDV) GP	Phase I	Safe and immunogenic	Bivalent	\	^257^
	ChAd3-Zaire GP	Phase III/IIa	Safe and immunogenic	Antibodies persisted to 1 year	\	^261,262^
	ChAd3-ZaireGP + MVA-BN-Filo	Phase I	Safe and immunogenic	Durable in immune response	\	^259,260^
Marburg virus	Ad5-GP	NHPs	100% protection	More immunogenic than DNA vaccine	Anti-vector immunity	^264^
Lassa virus	Ad5-GPC	NHPs	100% protection	\	Anti-vector immunity	
		Guinea pigs	100% protection	\	Poor NAbs	^265^
RVFV	ChAdO×1-Gn+Gc	Ruminants	100% protection	\	\	^268,270^
CCHFV	Ad5-N	Mice	S-specific Abs and NAbs	\	\	^266^
SARS-CoV-2	Ad5-S	Phase III	57.5% protection	Tolerable, safe in elder people,	Anti-vector immunity	^11^
	Ad26-S	Phase III	52.9% protection	/	Adverse effect	^12^
	Ad5-S + Ad26-S	Phase III	91.6% protection	Alleviate anti-vector immunity	\	^.^^15^
	ChAdOx1-S	Phase III	70.4% protection	/	Adverse effect	^13^
MERS-CoV	Ad5-S1/F/CD40 L	Mice	100% protection	Optimized immunogenicity	\	^542–544^
	ChAdO×1-S + MVA-S	Mice	100% protection	Long-term protection (40 week)	\	^545^
Hantavirus	Ad5-N/G_N_/G_C_	Syrian hamsters	100% protection	Nearly sterile protection	Poor NAbs	^546^

*RVFV* Rift Valley fever virus, *CCHFV* Crimean Congo hemorrhagic fever virus, *SARS-CoV-2* severe acute respiratory syndrome coronavirus 2, *MERS-CoV* Middle East respiratory syndrome coronavirus, *NHPs* nonhuman primates, *Abs* antibodies, *NAbs* neutralizing antibodies

#### Poxvirus vector

##### Heterologous prime-boost strategy combining poxvirus-vectored vaccines with other vaccine platforms

There have been several attempts to improve the immunogenicity of poxvirus-vectored vaccines, including the involvement of stronger promoters, deletion of genes responsible for immune regulation, and replacement of MVA181R/182 R with the anti-apoptotic gene B13R. Unfortunately, these attempts failed to achieve a breakthrough.^282–284^ Protective efficacy poxvirus-vectored vaccines could be ameliorated through a prime-boost strategy with other viral-vectored vaccine candidates. In a randomized clinical trial, a prime-boost regimen was conducted involving AD26-ZEBOV and MVA-BN-Filo.^285^ No serious vaccine-related adverse events were observed during vaccination and 8-month follow-up. The seroconversion rate of Ad26-ZEBOV recipients was higher than that of the MVA-BN-Filo group after primary vaccination. All vaccine recipients had detectable virus specific-IgG after booster with alternative vaccine and 8-month follow-up. Primed with Ad26-ZEBOV and boosted with MVA-BN-Filo elicited more vigorous cellular and humoral immune responses, which sustained for up to 1 year.^286^ The Ad26-ZEBOV + MVA-BN-Filo regimen was approved by the EMA on July 1, 2020.

Based on ChAdOx1 and MVA, a cross-filovirus immunogen was constructed based on conserved regions of the filovirus N, M and L protein. Protection of mice against Ebola and MARV was elicited by this vaccine candidate.^287^ In the absence of GP-specific antibodies and NAbs, ChAdOx1-MVA vectored prime-boost strategy elicited T cell immunity and conferred full protection, further demonstrating the prominent efficacy of heterologous prime-boost strategy.

For pathogens of complicated or multiple immunogen-dominant proteins, prime-boost strategy expressing different antigens is a promising strategy. Guillaume et al. developed a VACV-vectored vaccine expressing NiV glycoprotein G and fusion protein F, respectively, termed VV-NiV.G or VV-NiV.F.^288^ Two doses of VV-NiV.G or VV-NiV.F or a combined two-dose regimen induced binding antibodies and relatively low titers of NAbs in hamsters and provided 100% post-challenge protection. Moreover, the passive transfer experiment of immune serum proved that antibodies play an important role in the process of immune protection. Likewise, ALVAC-G and ALVAC-F were constructed using the canarypox virus (ALVAC)-vector.^289^ Although all protocols achieved full protection, pigs vaccinated with ALVAC-F showed low NAbs and a small amount of virus shedding, which was consistent with VSV-vectored NIV vaccine.^107^ Pigs vaccinated with both antigens (ALVAC-F/G) developed moderate neutralizing titers against HeV. The combined use of ALVAC-G and ALVAC-F induced the highest levels of NAbs and antigen-specific antibodies, which were likely to achieve sterile immunity. Virus shedding in pigs was also effectively blocked, indicating great significance in cutting off the NiV transmission chain from pigs to humans.

In Phase III clinical trials, ALVAC vectored HIV vaccine ALVAC-HIV was used alongside a recombinant glycoprotein 120 subunit vaccine. However, the protective efficacy was controversial.^290–293^

##### Expressing VLPs based on poxvirus vector

VLPs could mimic natural pathogens and render native presentation of antigens.^294–296^ Co-expression of VP40 and GP protein in EBOV resulted in the formation of EBOV-VLP and provided effective protection against challenge.^297–300^ Schweneker et al. developed MVA-BN-EBOV-VLP, in which VP40 and GP of EBOV Mayinga strain and NP of Tai forest virus Ebola were co-expressed based on MVA-BN vector.^301^ Human cells infected with MVA-BN-EBOV-VLP produced a large number of EBOV VLPs while poxvirus membrane protein B5 was excluded. MVA-BN-EBOV-VLP vaccinated mice produced EBOV GP-specific cellular and humoral immune responses quantitatively comparable to those of MVA-BN-EBOV-GP. Co-expression of GP and VP40 similarly led to the production of VLPs.^302^ However, no obvious advantage was observed in MVA-BN-EBOV-VLP vaccine candidates compared to MVA-BN-EBOV-GP in terms of immune response. Moreover, although full protection was achieved with 1 or 2 doses of MVA-VLPs vaccination, low transient viremia was detected in some vaccinated guinea pigs and NHPs.^303,304^ To protect against multiple pathogenic EBOV species, Karnail et al. developed a bivalent spherical Ebola VLP vaccine that incorporates GPs from ZEBOV and SUDV. Vaccination of rhesus macaques with bivalent VLPs generated strong humoral and cellular immune responses.^305^ The incorporation of both EBOV GP and SUDV GP significantly extended the breadth of both NAbs and ADCC responses compared to those of EBOV GP alone.

GEO-LM01, an MVA-vectored vaccine expressing LASV GPC and Z protein, could produce VLPs of LASV.^306^ A single IM dose of GEO-LM01 induced high levels of CD4^+^ and CD8^+^ T cells and provided 100% protection against LASV ML29 in mice.^306,307^ Co-expressing of ZIKV structural proteins PrM and E based on MVA resulting in the assembly of ZIKV VLPs. MVA-ZIKV VLPs induced potent NAbs and cellular immunity dominated by CD8 + T cell responses in mice.^308^ In addition, a single dose of MVA-ZIKV significantly reduced the viremia in susceptible immunocompromised IFNAR^−/−^ mice challenged with ZIKV.

##### Other poxvirus vectored vaccines in clinical

MVA-based CCHFV, MERS-CoV and SARS-CoV-2 vaccines have been constructed and moved into clinical trials.

A single dose of an MVA-vectored vaccine candidate expressing MERS-CoV S protein (MVA-MERS-S) induced high levels of NAbs in the mouse model. The immune response was dose-dependent. Histopathological analysis of lung and bronchial tubes showed that MVA-MERS-S limited replication of MERS-CoV in the lower respiratory tract of animal models.^309–311^ MERS-CoV is largely fueled by introductions from dromedary camels, thus evaluation of MVA-MERS-S was conducted in camels. After two doses of vaccination, MVA-MERS-S induced both systemic and local immunity in dromedary camels.^312^ The excretion of infectious virus and viral RNA was significantly reduced when challenged with MERS-CoV after two-dose immunizations. The protective effect was related to the presence of NAbs. In addition, vaccinated serum has cross-neutralizing activity against camelpox virus. The Phase I clinical trial showed that two doses of MVA-MERS-S inoculation induced antigen-specific antibodies and T cell responses in a dose-escalation manner. No serious adverse events were observed.^313^ The third dose of MVA-MERS-S boosted at 12 ± 4 months induced persistent MERS-CoV-S-specific B cells and antibodies for 2 years after the latest boost.^314^

MVA-SARS-CoV-2-S was constructed by expressing the full-length SARS-CoV-2 S protein based on the MVA vector. After two doses of vaccination, binding IgG antibodies and NAbs against SARS-CoV-2 were induced in mice, golden hamsters and rhesus macaques. After the SARS-CoV-2 challenge, vaccinated animals showed a significant reduction in viral loads, lung pathology, and free from symptomatic.^315–320^ IM, IN and IP delivery routes were all conducted and proved effective. However, point-to-point comparison and the delivery route-dependent manner of MVA-S in identical animal models were not performed. Therefore, it is difficult to determine the optimal delivery route. Representative Poxvirus vector-based vaccines for human disease were summarized in Table 9. Lentiviral vectors (LV), originally derived from human immunodeficiency virus (HIV), are ideal vaccine platforms due to their highly immunogenic and persistent immune responses even after a single dose immunization. The production of LVs involves the co-transfection of transfer vector, the envelope and packaging plasmids in appropriate cell lines. Pending that, the replication and integration of LVs can be abrogated by manipulation of the long terminal repeats (LTRs), the packaging signal, and the integrase gene.^321^ Apart from HIV, single-dose LV-based vaccines achieve progress in Zika and SARS-CoV-2 in pre-clinical studies, which have proven to be immunogenic, durable in immune response, and protective in challenge models.^322–324^ The Long-term immunity could be attributed to persistent transcriptions of LV in vivo. As reported, transgene expression could be detected in immunized mice and NHPs for 3–6-months post-injection or even longer.^324,325^ At cell level, LVs stay as an episomal form and produce the encoded protein for the lifetime of the cell, which raised the safety concerns about potential insertional mutagenesis with integrating vectors.^326^ Similar to the VSV/RABV ΔG strategy discussed above, anti-vector immunity of LVs can be coupled with a VSV GP serotype exchange strategy, even other envelope glycoproteins.^324,327,328^

**Table 9:** Vaccine candidates based on poxvirus vector

Virus	Design strategy	Stage	Results	Advantages	Overall concerns	Reference
Ebola virus	MVA-VP40 + GP + NP	Mice	Cellular and humoral immunity	Formulate VLPs	\	^301^
	MVA-GP + VP40	Mice	50–80% protection	Formulate VLPs	\	^302^
Lassa virus	NYBH-GP1 + GP2 + NP	NHPs	90% protection	\	Low Abs, safety concerns	^547^
	NYBH-GPC	NHPs	88% protection	\	Low Abs, safety concerns	
	MVA-NP	Mice and guinea pigs	100% protection	\	\	^548^
	MVA-GPC + Z	Mice	100% protection	Formulate VLPs	Low Abs	^306^
Rift Valley Fever Virus	VACV-vCOGnGc	NHPs	Immunogenic	NAbs higher than vCOGnGcγ	\	^549^
	VACV-vCOGnGcγ	NHPs	Immunogenic	Nearly sterile immunity	No protection efficacy in IFNAR^-^/^-^mice	
	rMVA-Gn/Gc	Mice	100% protection	\	No protective effect	^550,551^
		Lambs	Reduced virus shedding and viremia	\	Low effective	
CCHFV	MVA-GP	Mice	100% protection	\	Do not reduce virus load	^552^
	MVA-NP	Mice	Failed protection	\	\	^553^
SARS-CoV-2	MVA-S	NHPs	Protects from infection	Multiple immunation routes	\	^320^
MERS-CoV	MVA-S	Camels	Reduced virus load and viral RNA	Cross-neutralize camelpox virus	Existence of viremia	^312^
		Phase I	Humoral and cellular responses	Safe, well-tolerated	\	^554^
Zika virus	MVA-PrM E	Mice	Reduced virus replication	Cross-neutralization	Relatively low NAbs	^308^
Nipah virus	VV-G/F/G + F	Hamsters	100% protection	\	\	^288^
	ALVAC-G/F/G + F	Pigs	100% protection	Inhibition of virus shedding	\	^289^

*CCHFV* Crimean Congo hemorrhagic fever virus, *SARS-CoV-2* severe acute respiratory syndrome coronavirus 2, *MERS-CoV* Middle East respiratory syndrome coronavirus, *VLPs* virus like particles, *NAbs* neutralizing antibodies, *Abs* antibodies, *NHPs* nonhuman primates

#### Mucosal delivery of viral vectored vaccines induces local and peripheral immune responses

Mucosal delivery and triggering both local and systematic immune responses are extraordinary features of viral vector vaccines. Generally, mucosal delivery routes for vaccines include IN, IT, oral (OR), aerosol inhalation, etc. Intranasally or orally delivered VSV-vectored vaccines have been investigated. When rVSVΔG/ZEBOVGP vaccine was given via either IN, OR, or IM routes, NHPs were 100% protected against the lethal challenge of ZEBOV. The IN immunization of recombinant vaccines appeared to be more immunogenic than that of IM immunization.^88^ For COVID-19, immunization with rVSVΔG-S induced significantly higher NAbs and better post-challenge protection against SARS-CoV-2 through IN immunization than that of IM immunization in golden hamsters and NHPs.^329–331^ In human clinical trials, the antibody response following single IM dose administration of rVSV-SARS-CoV-2 was not ideal.^331,332^ This disappointing result may be related to the choice of vaccine delivery route. For respiratory disease, the VSV-vectored replication-competent COVID-19 vaccine could better simulate the natural infection process of SARS-CoV-2 through the respiratory tract, thus eliciting robust and protective immune responses. Tissue tropism and the expression and abundance of angiotensin-converting enzyme 2 (ACE2), the receptor of SARS-CoV-2, may help elucidate the delivery route-dependent manner of rVSV-SARS-CoV-2 in human and animal models. ACE2 is broadly distributed in the upper respiratory tract and lungs, while the distribution of ACE2 is low in skeletal muscle.^333,334^ Challenge studies in SARS-CoV-2 suspect animal models supported this hypothesis.^329,330^ Analogous results have been presented in the preclinical study of rVSV vector MERS vaccines.^114^

RABV vector was engaged as an OR-delivered bivalent rabies vaccine, known as rERAG333E, which contained a G333E mutation in the G protein of the RABV ERA strain.^335^ Subsequently, recombinant viruses rERAG333E/ZGP and rERAG333E/SGP expressing the glycoprotein of ZEBOV or SUDV were rescued.^336^ Both vaccines induced viral-specific NAbs and binding antibody responses in mice. However, rERAG 333E/ZGP induced lower ZEBOV NAbs than VSV-vectored Ebola vaccine either by OR or IM route.^337^ All rERAG333E/ZGP immunized dogs via OR route developed persistent NAbs against ZEBOV despite the pre-existence of anti-RABV immunity. Further, rERAG333E/NiVG and rERAG333E/NiVF were constructed based on rERAG333E vector expressing either attachment glycoprotein (NIV-G) or fusion glycoprotein (NIV-F) of NIV Malaysia strain.^338^ After OR immunization in mice and pigs, both rERAG333E/NiVG and rERAG333E/NiVF induced RABV and NIV NAbs and high levels of NIV-G or NIV-F specific Ig G. This study provided a safe and convenient OR vaccine for NiV for the first time. Therefore, live rERAG333E is a potential OR-delivered bivalent vaccine for free-roaming animals in endemic areas of RABV and other pathogens.

Mucosal vaccines based on PIV and NDV have been summarized in the second part of this review. These IN-delivered vaccines offer unique advantages for pediatric diseases and respiratory diseases.^165^ In addition, the preliminary application has been conducted in viral hemorrhagic fever, which appears to be less well effective than those for respiratory disease.^186–189^ Interestingly, PIV5‐G expressing RABV G induced protective immune responses via IN, IM, and OR immunization against lethal RABV challenge in mice, which was present as an efficacious paramyxovirus‐vectored OR rabies vaccine. It aligns with a needle‐free vaccination strategy to protect stray dogs and wild animals from rabies.^339^ Currently, mucosal-delivered IFV-vectored vaccines are limited to respiratory diseases. The limited size allowed for ectopic gene expression and concerns about stability hinder the rational application of IFV in other infectious diseases.

AdV vector vaccines are compatible with mucosal delivery due to distinctive tissue tropism, involving the upper or lower respiratory tract, gastrointestinal tract, or conjunctiva.^340,341^ Compared to IM immunization, IN administration of Ad5-EBOV provided complete protection of mice, guinea pigs, and NHPs from lethal challenge equally. Moreover, IN administration could bypass pre-existing immunity to Ad5 vector.^342–345^ In hamsters, the Ad5-vectored COVID-19 vaccine delivered orally or intranasally reduced disease severity and transmission.^346^ Heterologous boost immunization with an aerosolized Ad5-nCoV after two-dose priming with an inactivated COVID-19 vaccine is safe and highly immunogenic, and NAbs were significantly higher than that of homologous prime-boost strategy.^347–349^ ChAdOx1 nCoV-19 was also tested by IN-delivered in clinical trials. Mucosal responses to IN vaccination were detectable only in a minority of participants, which were largely lower than seen after SARS-CoV-2 infection. Systemic responses to IN vaccination were typically weaker than after IM vaccination with ChAdOx1 nCoV-19. Of interest, mucosal antibody was detectable in participants who received an IM dose of mRNA vaccine after IN vaccination.^350^ Vaxart developed an OR tablet COVID-19 vaccine consisting of an Ad5 vector expressing the SARS-CoV-2 S and N genes and RNA adjuvant. In Phase I clinical trials, the vaccine was well tolerated.^351^ Vaccine recipients exhibited an increase in mucosal secretory IgA that persisted up to 360 days. Nevertheless, no serum NAbs were observed. The protective efficacy against COVID-19 needs further investigation. The above results showed that mucosal vaccine may be an important supplemental pools for novel COVID-19 vaccines.^352^ Likewise, a single dose of ChAdOx1-S expressing MERS-CoV S protein immunization via both IN and IM routes induced a strong immune response and conferred protection against lethal challenge in lethal transgenic BALB/c mouse model.^353^

In the above proof-of-concept studies, IN or OR delivery of VSV-based vaccines performed well in viral haemorrhagic fever and beta coronavirus. IN delivery route was even more immunogenic than IM delivery. This may be owing to the rapid replication of VSV and abundant expression of antigens, simultaneously associated with the complicated pulmonary immune environment, which enabled massive antigen presentation and triggered both local and systematic immune responses. RABV vector was engaged as an OR-delivered rabies vaccine, which induced a long-lasting immune response. However, other mucosal delivery routes for RABV-based vaccines have not been fully investigated. PIV, NDV, and IFV-vectored vaccines were largely designed as IN-delivered regimens, particularly in respiratory disease. These single-dose vaccines were immunogenic and protective. Similar to those seen in VSV-based vaccines, IN administration of AdV-based EBOV vaccine provided better protection compared to IM immunization. This phenomenon further confirmed the potential of IN inoculation. However, as has been discussed, these viral vector-based vaccines were designed and delivered through different regimens and targeted separate antigens, rendering parallel comparison of mucosal delivery inapplicable. To our knowledge, potential of mucosal delivery may be tightly related to with the replication ability of the recombinant virus. In the near future, mucosal delivery of these viral vector vaccines warrants further investigation. Particularly, the correlation between mucosal immunity and protective efficacy should be clearly defined.

#### Application of viral vectors as therapeutic vaccines against cancer

**Fig. 2:**
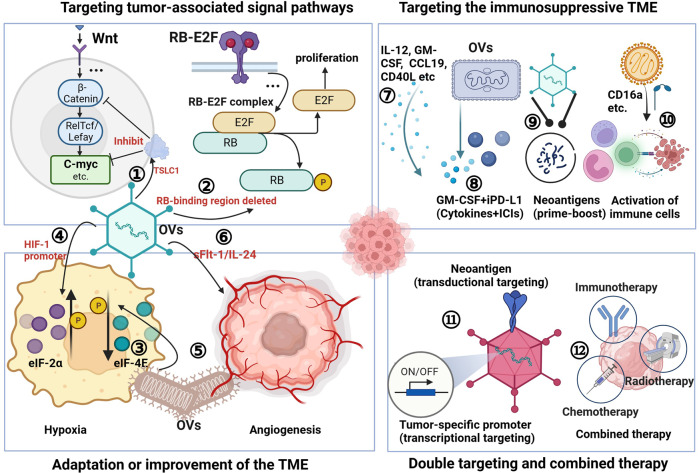
Mechanism for targeting and oncolysis of OVs. Oncolytic viruses (OVs) target dysfunctional signaling pathways. (1) Targeting the Wnt signaling pathway, recombinant adenovirus express lung cancer suppressor-1 (TSLC1), acts by downregulating the transcriptional activity of Tcf4/β catenin and inhibiting the expression of CyclinD1 and C-myc, thereby killing Wnt abnormally activated liver cancer cells. (2) E1A(922-947) deleted AdVs replicate and lyse tumor cells. OVs targeting for the adaptation or improvement of the tumor microenvironment (TME) of the tumor. (3) VSV replicates under hypoxic conditions with increased phosphorylated subunits of eIF-2α and inhibits the translation of host cell proteins through the dephosphorylation of translation initiation factor eIF-4E. (4) The Hypoxia inducible factors (HIFs) promoter was applied in AdV to the delivery target genes under hypoxia. (5) VSV naturally targets vascular endothelial cells and inhibits the growth of tumor cells. (6) Expression of vascular growth factor inhibitors (sFlt-1) and anti-angiogenic cytokine (IL-24) by OVs to inhibit tumor angiogenesis. OVs Targeting immunosuppressive TME. (7) OVs were designed to express a variety of cytokines, chemotactic factors, immune checkpoint inhibitors (ICIs) and neoantigens. (8) OVs simultaneously expressing cytokines (GM-CSF) and ICIs (iPD-L1). (9) Neoantigens encoding OVs and heterologous prime-boost strategy (AdV+MVA) (10) Activation of immune cells for tumor lysis by OVs. (11) Combine tumor-specific promoter-derived transcriptional targeting with transductional targeting (through viral capsid incorporation of anti-human carcinoembryonic antigen single variable domains). (12) Combined OV therapy with chemotherapy, radiotherapy and other immunotherapies, etc. (Created in BioRender)

Immunotherapy is an effective therapeutic approach in cancer, of which oncolytic virotherapy is an important branch of tumor immunotherapy.^354^ Briefly, oncolytic viruses (OVs) are naturally occurring or genetically engineered to preferentially replicat and selectively kill tumor cells, release TAAs, and stimulate the anti-tumor immune response through foreign gene delivery. Currently, four OV therapies have been approved worldwide, including Rigvir, Oncorine, Imlygic and Delytact, designed for melanoma, nasopharyngeal carcinoma, melanoma and glioma, respectively. Compared to prophylactic vaccines for infectious diseases, therapeutic cancer vaccines are more challenging as their activity may be hindered by consolidated immunosuppressive complicated tumor microenvironment (TME) and low immunogenicity of autologous TAAs. There are several aspects concerning the targeting and oncolysis process of OVs, including the surface receptors of the tumor cell, tumor-associated signaling pathways, TME, and anti-tumor immune cells (Fig. 2).^355–357^ Modification and manipulation of the genome of OVs enable the efficient targeting and killing of tumor cells, provided that the right tumor antigens are selected, in particular so-called tumor neoantigens.^358^

##### Targeting tumor-associated signaling pathways

The occurrence and progression of tumors are closely related to dysregulated intracellular signaling pathways. The Wnt signaling pathway is abnormally activated in tumors, mainly due to truncation mutation of colorectal adenomatous polyposis coli, render the formulation of stable β-Catenin, followed by the entry of β-Catenin into the nucleus, binding to Tcf/Lef family transcription factors and activate the cyclin D, C-myc and other Wnt target genes, which lead to tumorigenesis.^359^ RB-E2F is another signaling pathway concerning tumors.^360^ In normal cells, Retinoblastoma tumor suppressor protein (RB) inhibits E2F activity by recruiting histone deacetylation. When RB is dysfunctional, E2F releases and recruits transcriptional activators to promote the occurrence and progression of tumors. IFN signaling pathway is associated with antiviral immunity, simultaneously associated with tumors. IFN binds to the interferon receptor (IFN-R) and induces the expression of protein kinase R (PKR). When the dsRNA of virus binds to PKR, the activated PKR leads to the phosphorylation of elF-2α, which inhibits the protein synthesis, thus inhibiting the replication of the virus in cells.^361,362^ Post-entry of OVs, the cellular IFN response is a key determinant of oncolysis sensitivity. Gene expression signature has been devised to predict the outcome of oncolytic virus treatment designating constitutive IFN pathway activation.^363^

Genetic-engineered OVs are designated to act on these dysfunctional signal pathways. Targeting the Wnt signaling pathway, recombinant adenovirus Ad.wnt-E1A(Δ24bp)-TSLC1 expressing lung cancer suppressor-1 was constructed.^364^ Ad.wnt-E1A(Δ24bp)-TSLC1 could target and kill cells with abnormal activated Wnt signaling pathway, while showing no obvious killing effect on normal cells. Further study validated that TSLC1 down regulated the transcriptional activity of Tcf4/β catenin and inhibited the expression of CyclinD1 and C-myc, thereby killing Wnt abnormally activated liver cancer cells, which were further confirmed in the mouse xenograft tumor model of human hepatocellular carcinoma SMMC-7721.^365^ In the genome of AdV, 922–947 bp of E1A is the binding region of RB family. Thus, 922–947 bp of E1A was deleted to construct recombinant adenovirus dl922-947. Post infection with RB deficient tumor cells, dl922-947 can replicate and lyse tumor cells.^366^ What’s more, dl922-947 can effectively inhibit the occurrence of tumor metastasis in the xenotransplantation model of breast cancer. As reviewed, NS1 protein is a virulence factor of IAV, which can resist PKR mediated antiviral response in the IFN signaling pathway. NS1 deleted IAV cannot replicate in normal cells, while activated Ras can dephosphorylate PKR in tumor cells, thus NS1 deleted IAV can replicate in Ras activated tumor cells.^367^ Bergmann et al. knocked out the NS1 fragment of IAV to construct delNS1. Then delNS1 was tested in normal cells and normal cells expressing N-ras gene respectively. The results showed that delNS1 selectively replicated in normal cells expressing N-ras gene, which verified IAV as an effective OV targeting IFN signaling pathways.^368^ Therefore, targeting the abnormally activated signaling pathway can improve the targeting of OVs.

##### Targeting the adaptation or improvement of the TME

Hypoxia, neoangiogenesis, and immunosuppressive state are microenvironmental issues that determine the initiation, progression, and metastasis of tumors.^369^ Accordingly, OVs work by improving the hypoxic environment, inhibiting neoangiogenesis, and modulating the immunosuppressive state of the tumor, which correspondingly inhibit the proliferation and spread of tumor cells. Besides, the most common mechanism for tumor targeting of OVs is the handling of replication-associated genes, that is, inactivation of viral genes whose function is not required for replication in cancer cells, but is essential for virus replication in healthy tissues.

Coincidentally, VSV is more effective in mRNA production under hypoxia conditions.^356^ VSV can overcome increased phosphorylated subunits of eIF-2α under hypoxic conditions at the late stage of infection and inhibition of viral protein synthesis at the initial stage of infection. Meanwhile, VSV infection can inhibit host cell protein translation through the dephosphorylation translation initiation factor eIF-4E, which inhibits the growth and proliferation of tumor cells. Later, replication and cytopathic effects of VSV were confirmed in vitro and in vivo. Hypoxia inducible factors (HIFs) promoters can also be applied to target gene delivery under hypoxia. As the main molecule in the tumor hypoxia environment, HIFs are activated under hypoxia.^370^ Hypoxia inducible factor-1 (HIF-1) initiates transcriptional response under hypoxia conditions by directly binding to hypoxia responsive element (HRE). Therefore, HIF-1 and HRE genes are targets of tumor cells in a hypoxic environment. HYPR-Ad#1 is a modified AdV in which the E1A gene was under the HIF-1 promoter. HYPR-Ad#1 can only replicate in hypoxic tumor cells that show HIF-1 activation. After infection with HYPR-Ad#1 in HIF-1 activated brain tumor cells, E1A was overexpressed, and more than 90% of cells showed significant cytopathic effect (CPE). The above results indicate that HYPRAd#1 can be replicated in tumor cells activated by hypoxia and HIFs.^371^

Tumor angiogenesis plays a key role in the growth and metastasis of invasive tumors.^371^ Thus, inhibition of tumor angiogenesis through natural targeted killing effect or indirectly expressing vascular growth factor inhibitor may help inhibit tumor angiogenesis. VSV can naturally target vascular endothelial cells. Infection with VSV can effectively inhibit the growth of tumor cells. Previous research has shown that wild-type VSV specifically infected endothelial cells (ECs) in tumor tissues and showed anti-tumor effect.^372^ In addition, expression of vascular growth factor inhibitors by OVs could improve their ability to inhibit tumor angiogenesis, thereby inhibiting tumor growth and metastasis. ZD55-sflt-1 is an oncolytic AdV expressing soluble vascular endothelial growth factor receptor inhibitor sFlt-1. It showed an inhibitory effect on tumor angiogenesis in the animal model of human colorectal cancer.^373^ Specifically, IL-24 is an effective anti-angiogenic cytokine, which can inhibit angiogenesis and induce apoptosis of tumor cells.^374^ IL-24 gene was inserted into AdV, termed HE1B55D-RGD-IL-24.^375^ On this basis, the additional anti-angiogenic arrested fragment was inserted into AdV to construct HE1B55D-RGD. After administration of these two oncolytic adenoviruses in the nude mouse melanoma transplant tumor model, vascular endothelial growth factor (VEGF) and transforming growth factor β were inhibited, which led to anti-angiogenic activity.

Oncolytic efficacy could be further activated by tumor-secreted matrix metalloproteinases (MMP). Originally, MeV fusion protein encompasses a furin cleavage site and depends on intracellular proteases to process proteins and activate particles. Thus, the replacement the furin cleavage site with sequences recognized by matrix metalloproteinases or the urokinase-type plasminogen activator increased tumor specificity, that is, recombinant MVs expressing the modified F proteins spread only in cells secreting MMP. These studies emphasized the conjunction between the targeting and particle activation of OVs under the tumor microenvironment.^376,377^

##### Targeting the immunosuppressive TME

Cancer cells adapt to tightly restrict anti-tumor immunity by expressing multiple inhibitory ligands, which serve the so-called ‘immune checkpoint’ molecules for immune cells, thus delivering inhibitory signals that block T cell activation and survival.^378^ To regulate the immunosuppressive state and enhance anti-tumor immunity, OVs were designed by expressing a variety of cytokines, chemotactic factors and immune checkpoint inhibitors (ICIs) and acting jointly with chimeric antigen receptor T cells (CAR-T).^379,380^ Besides, neoantigens are supposed to be promising tumor antigens for cancer vaccination with no self-tolerance but the potential to induce robust and selective T cell responses. The discovery and use of neoantigens depends on new technologies such as next-generation sequencing. The definition of tumor-specific neoantigens together with the approval of effective ICIs, contributes to the clinical development of novel vector-based cancer vaccines.

OVs expressing cytokines such as IL-12, IL-13, granulocyte-macrophage colony-stimulating factor (GM-CSF), chemotactic factor CCL19, and CD40L have been proved to effectively recruit and activate antigen presenting cells (APCs) and CD4^+^T cells and CD8^+^T cells and reduce the expression of TGF-β and VEGF, thus alleviating the immunosuppressive.^381–386^ Notably, interleukin-13 receptor α2 (IL-13Rα2) was overexpressed in 80% of glioblastoma multiforme (GBM) tumors. To retarget GBM via IL-13Rα2, MeV-GFP-H_AA_-IL-13 was generated by displaying human IL-13. MV-GFP-H_AA_-IL-13 exhibited potent syncytia formation in IL-13Rα2 overexpressing glioma lines. In vivo treatment of MV-GFP-H_AA_-IL-13 significantly prolonged survival in orthotopically implanted GBM12 xenograft mice. Neurotoxicity was not observed post administration of MV-GFP-H_AA_-IL-13 in the central nervous system in mice.^386^ Vaccinia virus VV-IPDL1/GM was constructed by simultaneously expressing GM-CSF and programmed death-ligand inhibitor (iPD-L1). In tumor cells, iPD-L1 binds to programmed death-ligand (PD-L1), thus restoring anti-tumor cell immunity. Intratumoral administration with VV-IPDL1/GM in B16-F10 mouse melanoma model may promote the maturation of tumor infiltrating dendritic cells (DCs) and the activation of tumor-specific T cells. VV-IPDL1/GM provides a more effective targeted therapy regimen for targeted tumor therapy, particularly for patients with resistance to PD1/PDL1 blocking therapy.^387^

Moving away from the use of canonical immune mediators, the clearance of immunosuppressive molecules from the TME has also been considered. An oncolytic VACA was engineered to express the prostaglandin-inactivating enzyme hydroxyprostaglandin dehydrogenase 15-(NAD) (HPGD). Expression of HPGD selectively depleted Treg and myeloid derived suppressive cells (MDSCs) populations, thereby enhancing the antitumor immune response by upregulation of Th1-associated chemokines.^388^ Similarly, metabolic reprogramming of TME has been achieved by engineering oncolytic VACA to express leptin. Leptin, in the context of OV-induced immune infiltration, improved mitochondrial oxidative phosphorylation (OXPHOS) in tumor infiltrating lymphocytes, preventing metabolic exhaustion of CD8 + T cells and thus enhancing the therapeutic efficacy and antitumor memory development.^389,390^

MY-NEOVAX, an oncolytic adenoviral platform encoding up to 50 patients’ specific neoantigens, has been developed. MY-NEOVAX therapy proved effective in improving survival in two patients with last-line treatment refractory colorectal cancer and high-grade neuroendocrine carcinoma.^391^ Based on AdVs derived from non-human Great Apes, the potency and efficacy of a novel Great Ape Adenoviral (GAd) encoding multiple neoantigens was investigated.^392^ Prophylactic or early therapeutic vaccination with GAd efficiently control tumor growth in mice. In contrast, combining the vaccine with checkpoint inhibitors is required to eradicate large tumors. Abundance of activated tumor infiltrating T cells with a more diverse TCR repertoire in animals treated with GAd and anti-PD1 compared to anti-PD1. This study suggests that vaccination effectiveness in the presence of a high tumor burden correlates with the breadth of neoantigens-specific T cells and requires concomitant reversal of tumor suppression through checkpoint blockade. Heterologous prime-boosting strategies have also been applied in tumor vaccines to overcome the anti-vector immunity. Leoni et al. has developed a neoantigen-based prime-boost vaccine for the treatment of microsatellite instability (MSI) tumors.^393^ Neoantigens (FSP) were selected and cloned into non-human GAd and MVA vectors to generate a virus-vectored vaccine, referred to as Nous-209. In mice, Nous-209 was potent to induce broad FSP-specific CD8^+^ and CD4^+^ T-cell responses. Moreover, FSP was processed in vitro by human APCs and subsequently human CD8^+^ T cells were activated. Nous-209 encodes many neoantigens who shared across MSI tumors, which induces the optimal breadth of immune responses, which may achieve clinical benefits to treat and prevent MSI tumors. Another heterologous prime/boost regimen based on non-replicating AdVs combined with oncolytic Maraba virus MG1, both expressing MAGE-A3, has been shown to induce the expansion and long-term persistence of TAA-specific immune response in Macaca.^394^ These results indicated that the heterologous prime-boost strategy was equally applicable in cancer therapy.

##### Transcriptional and transductional double targeting

In theory, targeting tumor cells via surface receptors based on the natural tropism of OVs or genetically modified OVs is applicable. However, this receptor-based strategy is not suitable for OVs with extensive receptors and cell tropism, which hinders the precise killing of tumors. By engineering TAA-specific ligands (or antibodies) on virus particles, retargeting OVs to tumor-specific antigens was achieved.^395,396^ The full replication activity of OVs was maintained without compromising their safety profile.

To further improved the precision of targeting, attempts have been made to combine tumor-specific promoter-derived transcriptional targeting with transductional targeting (through viral capsid incorporation of antihuman carcinoembryonic antigen single variable domains).^397^ The results showed that employment of a single variable domain genetically incorporated into an AdV fiber increased specificity of infection and efficacy of replication of single variable domain-targeted oncolytic AdV. Double targeting, both transcriptional and transductional, is a promising means of improving the therapeutic index for these advanced generation conditionally replicative AdVs. This re-targeting strategy provides selectivity for OVs to tumor cells, simultaneously enabling the de-targeting of the virus from its natural receptor. This approach is considered suitable for systemic delivery as it minimizes virus replication in healthy tissues.^394^

##### Activation of immune cells

MeV naturally and preferentially replicates in malignant cells, which facilitates antitumor immunity and tumor lysis.^398–401^ To improve the cytotoxic activity against tumor cells directed by MeV-activated NK cells, oncolytic MeV vaccines encoding both CD16A on NK cells and carcinoembryonic antigen (CEA) as a model tumor antigen was developed, termed bispecific killer engagers (MV-BiKE).^402^ MV-BiKE mediated the secretion of functional BiKE from infected tumor cells. In colorectal or pancreatic cancer cells, MV-BiKE specified the anti-tumor cytotoxicity by NK cells and mediated expression of effector cytokines and degranulation. Viral vector vaccine-harnessed NK cells as anti-tumor effectors were proved in this proof-of-concept study. Analogously, OVs can also increase myeloid and plasmacytoid DCs-mediated cytotoxicity, modulate macrophages toward an antitumor phenotype and activate neutrophils, leading to secretion of related cytokines.^403,404^

NDV has been reported as OV in clinical. Pediatric high-grade glioma was treated with the oncolytic viral MTH-68/H, an attenuated strain of NDV, combined with oral valproic acid. The above treatment resulted in a far-reaching regression of thalamic glioma despite second neurosurgical intervention were required subsequently. This study documents the oncolytic effect of NDV directed toward virus presence and replication in neoplastic cells.^405^ In Phase I/II trial in patients with GBM, oncolytic NDV was well tolerated when high doses were applied and responsible through intravenous delivery.^406^

##### Advanced OVs in clinical and approved OVs

Herpes simplex virus type 1 (HSV-1) is a distinguished OV work through intratumoral replication and induction of antitumor immune responses.^407^ Talimogene laherparepvec (T-VEC, Imlygic) is an HSV-1 based OVs for unresectable stage IIIB–IV melanoma.^408^ Underwent preclinical evaluation in cell lines and animal models,^409–411^ T-VEC was extensively evaluated in clinical trials. In the Phase I clinical trial, HSV-1 was engineered to express GM-CSF, another mechanism for enhancing local and systemic anti-tumor immunity was aroused by recruitment and maturation of dendritic cells.^412^ Further, the efficacy of T-VEC was evaluated in Phase II clinical trials.^413^ In 50 participants with advanced-stage melanomas, 10 patients had a complete response (CR) and 3 patients had a partial response (PR) following a median of six injections of T-VEC, the overall response rate (ORR) was 26%. The overall survival rate was 58% at 1 year and 52% at 24 months, these evidences proved the systemic effectiveness of T-VEC. T-VEC ameliorated durable response in patients with advanced melanoma in Phase III clinical trials. The durable response rate was significantly higher in T-VEC treatment group (16.3%; 95% CI, 12.1–20.5%) compared with GM-CSF treatment group (2.1%; 95% CI, 0–4.5%); odds ratio, 8.9; *P* < 0.001). The ORR and overall survival rate were also superior in T-VEC treatment group, with most pronounced therapeutic efficacy in patients with stage IIIB, IIIC, or IV melanoma. T-VEC remains the only widely approved OV therapy, which has been optimized during the clinical application process.^414^

G207 is the second-generation oncolytic HSV-1 involves deletions in the γ34.5 gene and inactivation of the ICP6 gene.^415^ These modifications diminished pathogenicity and promoted anti-tumor properties.^416^ Besides direct oncolytic activity, G207 was proven to strengthen anti-tumor immunity in a mouse tumor model.^417^ In patients with malignant glioma, G207 was safe when applied pre-and post-tumor resection and in combination with other tumor therapy.^418–421^ During the evolution process, the deletion of alpha47 gene and overlapping of the promoter region of US11 from the second-generation oncolytic HSV-1,^415^ enhanced the tumor-specific replication capability and cytopathic effect in tumor cells.^407,422,423^ This third-generation HSV-1, namely G47∆, was significantly more efficacious in vivo than its parent G207 at inhibiting tumors while maintained safety profile. In Phase I/II clinical trials, G47Δ was administered for up to six doses or two doses within 2 weeks in patients with residual or recurrent glioblastoma. G47Δ treatment was associated with improved survival rate and median overall survival. Overall, response and stable disease in patients were observed during the follow-up.^424,425^ Biopsies revealed that the TEM was improved by increased numbers of tumor-infiltrating lymphocytes. G47∆ (Delytact) has been approved in Japan for glioblastoma.

H101 is an E1B-55 kDa gene-deleted replication-selective AdV, which has been approved as an OV in China (Oncorine). In a Phase III randomized clinical trial, H101 was applied for the treatment of nasopharyngeal carcinoma. H101 in combination with chemotherapy achieved an ORR of 72.7%, compared to that of 40.3% with chemotherapy alone.^426^ Overall, H101 was safe and effective in patients with squamous cell cancer. Similarly, AdV-based OVs, DNX-2401, has been tested in clinical trials for recurrent malignant glioma, which resulted in potent responses and long-term survival, which may owe to direct oncolytic effects of the virus, followed by elicitation of an immune-mediated anti-glioma response.^427,428^ Nadofaragene firadenovec (rAd-IFNa/Syn3) is a replication-deficient rAdV that delivers human interferon alfa-2b cDNA into the bladder epithelium, which was indicated for Bacillus Calmette-Guérin-unresponsive non-muscle-invasive bladder cancer.^429^ Post-treatment with Nadofaragene firadenovec, 53.4% (55/103) of patients with carcinoma in situ had a complete response within 3 months of the first dose and this response was maintained in 45.5% (25/55) of patients at 12 months. The above results suggested that nadofaragene firadenovec was efficacious and provided favorable benefits.

ECHO-7 (Rigvir), an approved echovirus for stage I–II melanoma, decreased the risk of disease progression with ECHO-7 relative to other experimental immunotherapies.^430^

##### Concerns and prospects of OVs

Natural viruses are applied as the first generation of oncolytic virotherapy. Although they achieved some efficacy in the treatment of solid tumors and a small number of metastatic tumors, they suffered from defects such as poor targeting, side effects, inability to elicit effective tumor immunity, and the ability to be administered intratumorally only. Further, improved tumor targetability, reduced toxic side effects and boosted antitumor immunity through the insertion of cytokines, etc. have been pursued the treatment of refractory solid tumors and metastases.^431^ Although oncolytic virotherapy has made tremendous progress, there are also many obstacles in the therapeutic process. Beyond these issues appeared in prophylactic viral vector vaccines, like anti-OV immunity, the transgene stably etc.,^380^ both the route of administration of OVs and the choice of clinical patients will be difficult during the development of OVs. Although some of the above issues could be addressed by equivalent solutions in prophylactic viral vector vaccines, additional countermeasures are needed.

To further improve the therapeutic efficacy of OVs, remarkable breakthroughs have been made in combination therapy with oncolytic virotherapy T-VEC, anti-PD-1 antibody and chemotherapies.^432^ In the treatment of melanoma, T-VEC combined with PD-1 antibodies resulted in a tumor objective response rate of up to 62%, with a complete response rate of 33%.^433^ For patients with resistance to antibody therapy, treatment with Oncorine together with antibody drugs provides additional benefits. The patient experienced symptomatic improvement and achieved stable disease despite partial necrosis of lung tissue.^434^ These findings suggest that oncolytic virotherapy combined with tumor-associated antibodies and chemotherapy drugs may perform better by altering the tumor microenvironment.

Nonetheless, the correlation between the immune response induced by OVs and antitumor efficacy is largely unknown, either innate or adaptive anti-tumor immunity. Clinical knowledge of the combination of OV and CPI will help to better understand how to optimize the use of these viruses for cancer therapy. Of importance, the in-depth understanding and accurate harness of the underlying biology and pharmacology of OVs may enable the systemic administration of OVs and broaden their range of application in cancer.

### Several key aspects of viral vector vaccines

#### Trigger immune responses in a delivery route-dependent manner

**Fig. 3:**
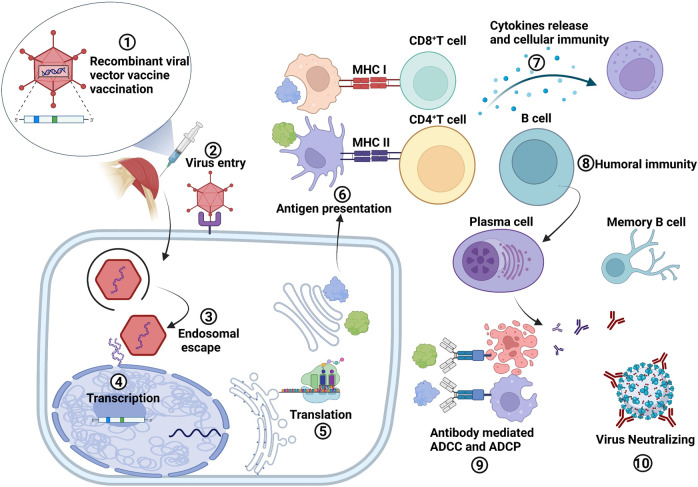
Antigen presentation mechanisms of intramuscular-delivered viral vector vaccine. (1) Construction and vaccination of a recombinant viral vector vaccine. (2) Viral vector vaccine is taken up by muscle cells through endocytosis. (3) Endosome escape from viral vector vaccine. (4) Partially disassembled virus capsid traffic to the nucleus, initiating the transcription process. (5) Protein translation and post-translational modification in endoplasmic reticulum and golgi apparatus. (6) Antigen proteins are presented to antigen presenting cells. The antigen can be loaded onto MHC class I for direct presentation to CD8^+^ T cells, and loaded onto MHC class II for direct presentation to CD4^+^ T cells. (7) T cells lyse infected cells under the mediation of cytokines. (8) Stimulated B cells differentiate into memory B cells and antibody-releasing plasma cells. (9) Antigen proteins are recognized by antibodies, including those capable of Fc-mediated effector function, including antibody-mediated cellular cytotoxicity and antibody-dependent cell-mediated phagocytosis. (10) Neutralizing antibodies neutralize pathogens and block their entry. (Created in BioRender)

The clarification of immune response triggered in delivery routes-dependent manner is of significance to vaccine design and delivery route selection for specific pathogens. The potential mechanism of antigen presentation and immune response induction post-IM vaccination of the viral vector vaccine was illustrated in Fig. 3 (Recombinant AdV as an example).^435^ Virus entry into muscle cells is mediated by receptor and ligand recognition. Then viruses are absorbed through endocytosis. In cytoplasm, the virus escapes from the endosome, partially disassembles capsids and enters the nucleus through the microtubule network. Transcription of the target genes is conducted in the nucleus, then translation and post-translational modification of the antigen protein are completed in the endoplasmic reticulum and golgi apparatus, respectively. The capture of antigen proteins by APCs resulted in MHC class I presentation and MHC class II presentation to CD8^+^ T cells and CD4^+^ T cells, respectively. CD8^+^ T cells mediate cytolysis of infected cells under the regulation of cytokines. Meanwhile, stimulated B cells differentiate into memory B cells and plasma cells. Plasma cells produce NAbs and binding antibodies. These antibodies are involved in virus neutralization and Fc mediated function, including ADCC and ADCP, etc.

**Fig. 4:**
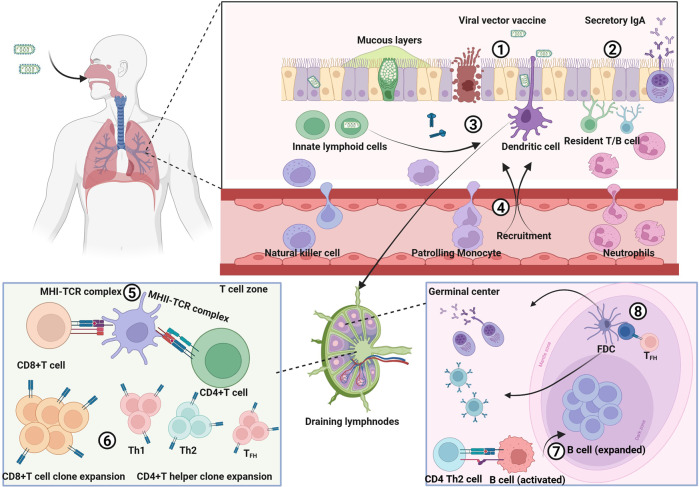
The local and peripheral immune response induced by intranasal-delivered viral vector vaccine. (1) Intranasal delivery of the viral vector vaccine and entry of the recombinant virus. (2) Local secretory immunoglobulin A produced by subepithelial plasma cells. (3) Antigen is recognized and processed by innate immune cells and antigen presenting cells. (4) Immune cell recruitment, including neutrophils, natural killer cells, and monocytes. (5) Activated dendritic cells traffic to draining lymph nodes via afferent lymphatics to prime adaptive responses. In lymph node T cell zones, externally derived antigens are presented on class II MHC, prompting CD4^+^ T cell training, while internally derived antigens are processed and presented on class I MHC to CD8^+^ T cells. (6) APCs promote maturation and expansion of naive CD4^+^ and CD8^+^ T cells. CD8^+^ cytotoxic T cells and subsets of CD4^+^ T helper cells traffic back to the site of infection. (7) Activated B cells undergo expansion and somatic hypermutation of the B cell receptor (BCR), resulting in BCR specificity that strongly binds to peptides maintained on the surface of follicular dendritic cells (FDCs). B cells cycle through iterative rounds of expansion/somatic hypermutation and affinity selection, resulting in the selection of high-affinity BCRs. (8) Interactions with T follicular helper (Tfh) cells lead to B cell differentiation and class-switching to long-lived memory B cells and high-affinity plasma cells, which traffic to sites of infection or maintained as long-lived memory populations. (Created in BioRender)

In the unique circumstances of the respiratory tract, innate and adaptive immune responses are tightly regulated and in continual flux for careful balance between pathogen clearance, immune modulation, and tissue repair.^436^ Compared to IM delivery, IN delivery of recombinant viral vector vaccine induces both local and peripheral immune response (Fig. 4) (rVSV as an example). After vaccination and virus entry into the mucosa, secretory immunoglobulins (sIgA and sIgM) are produced by subepithelial plasma cells. They provide antigen-specific targeting of foreign antigens parallel to their innate immune counterparts. Simultaneously, innate immune cells are recruited. Some of them process and pass the antigen to APCs, mainly DCs. Activated DC traffic to drain lymph nodes (LNs). In the T cell zone, DCs train naive T cells and lead to clonal expansion. Then, antigen recognition induces effector expression by T cell activating B cell. Activated B cells enter the germinal center (GC), undergo expansions, leading to long-lived memory B cells and high-affinity plasma cells.^436^

**Fig. 5:**
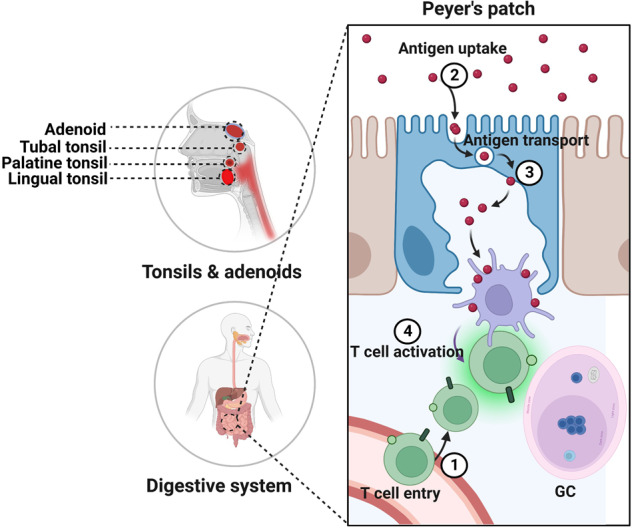
Potential sites for induction of immune response induced by oral-delivered viral vector vaccine. In the oral cavity, viral vector vaccines enter through oral lymphoid tissue, including adenoid, tubal tonsil, palatine tonsil, and lingual tonsil, etc. In the small intestine, Peyer’s patch is the core site for immune response (1) T cells enter Peyer’s patch from blood vessels. (2) M cells take up antigens by endocytosis and phagocytosis. (3) Antigens are transported across the M cell in vessels and released at the basal surface. (4) T cells in the Peyer’s patch encounter antigens and are activated by dendritic cells. (Created in BioRender)

Several vaccine candidates have shown potent profile post OR vaccination.^88,337,338,347,348,351,437^ Nevertheless, limited data on the interaction between OR-delivered recombinant virus vector vaccine and the complicated oral gastrointestinal (GI) environment rendered the mechanism unknown in terms of virus entry and establishment of humoral and cellular immune responses at both systemic and mucosal sites. Current evidence shows that tonsils & adenoids as well as Peyer’s patch in the small intestine are potential sites for induction of post-vaccination immune response (Fig. 5).^438,439^ Notably, Peyer’s patches are core sites for immune response stimulation. This process involves antigens uptake by M cells, transport and release of antigens, and activation of T cells. However, due to the harsh chemical conditions in the stomach and intestine, it seems difficult for viral vector vaccines to reach the small intestines without the package of specific material. In the near future, antigen presentation mechanisms of viral vector vaccine post OR inoculation warrant further investigation.

#### Choice of viral vector platforms and balancing safety and immunogenicity

Safety and immunogenicity are key components of a promising vaccine. In most cases, a delicate balance should be achieved depending on the given condition. For urgent large-scale vaccination against lethal haemorrhagic fever with a high fatality rate, rVSV-vector replication-competent vaccine is a reasonable choice since its characterization of a single-dose regime, robust immunogenicity and rapid immune response. For frequently emerging respiratory diseases such as influenza and COVID-19, PIV, IFV and NDV-vectored vaccines provide eligible options for IN-delivered, single-dose vaccine or booster vaccines. For medical workers with corresponding medical conditions, multi-dose inoculation of RABV-based inactivated vaccines or heterologous prime-boost regimen based on AdV and poxvirus vectors combine immunogenicity and durability while minimizing anti-vector immunity. The heterologous prime-boost regimes could maximize the benefits and circumvent the limitations of those seen in specific single vectors. In the near future, clearer definitions of the general and distinctive characteristics of these viral vectors are needed to further support the selection of viral vectors.

According to the limited study about the point-to-point comparison of these NNSV vector vaccines, VSV vector vaccines appear to be more immunogenic and effective after a single dose of IM inoculation than RABV or PIV and DNA virus vector vaccines. The above phenomenon can be attributed to the robust replication dynamics of VSV. As a live vector, PIV is considered to be a relatively safe vaccine vector, which is advantageous over other NNSV vectors developed from which encounter issues with virus reversion, residual virulence, etc. In particular, the safety of the PIV vector vaccine has been assessed in children over 2 months, which represents an ideal platform for pediatric diseases.

Indeed, replication-competent viral vector vaccines provide additional benefits for mucosal delivery and the duration of immune response. In contrast, strong replication ability may be followed by a higher risk of adverse effects, especially in immunocompromised individuals like pregnant women, infants and the elderly. Therefore, an attenuation strategy is needed to address the biosafety issue.^440^ Ideally, attenuation of the viral vector vaccine should maintain immunogenicity. For VSV, the second generation rVSV design strategy was implemented, termed N4CT1, which involves an additional transcriptional unit at the 3′ end of the genome, translocation of the N gene to the fourth transcriptional unit, and truncation of the VSV G CT domain (Fig. 1a).^124^ N4CT1 is attenuated by changing the gene location of specific proteins in the genome, which has been verified in human clinical trials. This strategy may also apply to other NNSVs. Besides, viral mRNA cap (methyltransferase, MTase) activity is an excellent target for the development of live attenuated viral vector vaccines, as the viral mRNA cap is essential for mRNA stability, protein translation, and innate immune evasion.^441–444^ Deficiency of MTase has been shown to completely attenuated in both immunocompetent and immunocompromised mice while the immunogenicity was not dampened. In addition, utilizing a temperature-sensitive assembly-defective mutation of L111A and combining it with an M51R mutation in the M protein of rVSV significantly reduced the pathogenicity of the virus while maintaining highly effective virus production.^32,445^ These strategies can be stand alone or combination to improve the safety of replication-competent viral vector vaccines. In RABV, previous efforts in the attenuation strategy were directed towards the deletion of pathogenic genes (G/P/M).^446–454^ Nevertheless, these strategies rendered recombinant virus replication-defective, thus compromising post-challenge protection effectiveness, making them less attractive. Instead, the attenuation strategy was conducted based on SADB19, a vaccine strain licensed in Europe for wild animal vaccination.^455–457^ RABV SADB19 involving an R333E mutation in G protein was proved to be significantly attenuated in neurovirulence.^458^ Similarly seen in PIV vector vaccines, over-attenuation may lead to suboptimal efficacy in humans.^175,179^ Consequently, pending the attenuation of viral vectors, immunogenicity should be timely regarded and maintained.

Currently, there have been three generations of AdV vectors. The first generation AdV vector lacks E1 or E3 genes. This type of vector can cause strong inflammatory response and immune responses. In the second generation AdV vector, the E2A or E4 gene was further deleted, resulting in a weaker immune response, but improved capacity and safety. The third generation AdV vector lost all or most of the AdV genes, retaining only inverted terminal repeat (ITR) and packaging signal sequences. The cellular immune response caused by the third generation AdV vector is further reduced. The evolution of AdV vector is also a balancing process between safety and immunogenicity.

To sum up, the selection of viral vectors, replication-competent, single-round replication or inactivated, is a balance between safety and immunogenicity, and depends on the properties of given pathogens and the target population.

#### Mucosal delivery is a prominent feature of viral vectored vaccines

Due to the intrinsic adjuvant properties and active mucosal infection, viral vector vaccines could be delivered via mucosal routes and offer several distinguished advantages. (1) Beyond the systematic immune response, local mucosal immune response induced by mucosal vaccines would serve the first line of defense against foreign pathogens, which is supposed to block virus entry and provid broader heterosubtypic protection.^459–467^ As has been reviewed, humoral immune responses in PBMCs are not always the exclusive indicator for evaluating a mucosal vaccine.^180,238^ For instance, the secretory immunoglobulin A (SIgA) in the nasal cavity can last for about 9 months after natural infection with SARS-CoV-2, whereas injectable vaccines are effective in producing and enhancing antibodies in the blood, and can prevent serious diseases, but have little impact on nasal IgA levels.^468–470^ (2) Local CD8^+^ T cells and SIgA exhibit broader spectrum effects than NAbs, which would be particularly essential for frequently mutated pathogens like IFV and SARS-CoV-2.^471,472^ (3) Mucosal immunity could alleviate the impact of anti-vector immunity on viral vector vaccines to some extent.^342^ (4) These mucosal immunization routes are more convenient and acceptable than injectable vaccines, especially for needle-fearing populations, which would contribute to the full establishment of herd immunity. Simultaneously, for diseases of animal origin, mucosal-delivered vaccines are convenient and practicable. IN or inhalation inoculation could achieve large-scale immunization in huge animal groups whilst OR inoculation facilitates the full distribution of vaccines in wildlife habitats. (5) In cases that most people worldwide have received at least two doses of injectable COVID-19 vaccines, either mRNA vaccines or inactivated vaccines, boosting with mucosal-delivered viral vector vaccines would consolidate the systemic immune response and offer additional mucosal immune response.^347^ (6) Mucosal vaccines may help fill gaps in traditional vaccines.^473,474^ For example, results from numerous clinical trials of licensed SARS-CoV-2 vaccines have shown lower efficacy in older adults than in younger adults.^11,475,476^ While dNS1-RBD, an IN-delivered COVID-19 vaccine, was well tolerated in all participants aged 18–86 years, and immunogenicity in older adults (aged ≥60 years) was similar to that in younger participants. (7) For those mucosal-associated pathogens that transmit through the respiratory or digestive tract, viral vector vaccines could maximize the recapitulation of the natural infection process of specific pathogens. Consequently, provide a comprehensive immune response and protection. (8) The respiratory tract and digestive tract are not completely separated. For example, Ad5­nCoV is administrated by aerosol inhalation through the oral cavity, which is then fully distributed in the respiratory tract, mainly in the lungs.^347^ That is, the IN-delivered vaccine could be converted into an oral-respiratory aerosol inhalation vaccine, as the inhalation vaccine provides better immune response and protection than nasal spray vaccines.^477^

Nevertheless, the relationship between local mucosal immunity and protective efficacy is not yet well established, especially in human clinical trials. Translational gaps between animals and humans should be noted. Promising results in preclinical animal studies may not necessarily predict safety and efficacy in humans. The human immune system is more sophisticated, and the local environment of the human nasal or respiratory tract is likely to have been exposed to a variety of pathogens prior to trial participation, whereas that of an animal raised in a controlled laboratory environment is likely to be naive to such exposures, which may affect immune responses to vaccination. This gap between species should be further explored. Besides, for the evaluation of those replication competent viral vector vaccines assembling solely foreign glycoprotein, the animal model should strictly reflect the actual situation in humans in terms of receptor-ligand recognition, pre or post exposure, and composition of the immune system.^329,330,478^

More recently, novel vaccine technologies such as mRNA vaccines and protein subunit vaccines attract attention and are also involved in mucosal vaccine platforms.^479^ For mRNA vaccines, intranasally administered COVID-19 mRNA vaccines systemically induced S-specific binding antibodies and NAbs comparable to IM inoculation group.^480^ Correspondingly, IN vaccination exhibited protective efficacy against challenge of SARS-CoV-2 in hamsters. Nevertheless, secretory IgA in the turbinate and alveolar lavage fluid was not detected in this study, thus the local immune response and activation of tissue resident T or B cells were uncertain. For the protein subunit vaccine, a vaccine strategy called “prime and S” was noted,^352^ which was conducted by boosting IM-delivered COVID-19 mRNA vaccines with IN-delivered S protein vaccines. Robust resident memory B and T cell responses and IgA were induced in the respiratory mucosa of mice. Actually, this strategy aroused mucosal immunity by protein vaccine on the condition that existing immunity was generated by primary vaccination, which elicited mucosal immune memory in the respiratory tract. In theory, all vaccine approaches could be conducted likewise the “prime and S”. Overall, preclinical data concerning mucosal vaccines are limited for mRNA vaccines and protein subunit vaccines. To a large extent, local mucosal immunity induced by solely mRNA vaccines is uncertain. Pre-existing immunity is required for mucosal delivery of protein subunit vaccines. In contrast, viral vector platforms were well established in mucosal vaccines, which induced both local and systemic immune responses ignoring the immune status. Importantly, innate myeloid cells, such as monocytes/macrophages, can produce vigorous responses following subsequent encounters, so called “natural immune memory” or “trained immunity”, which may provide support for viral vector vaccine in mucosal delivery.^481–483^ As has been reported, respiratory virus infection simulated alveolar macrophage memory and produced trained immunity, fosterting a sustained response to a secondary challenge, this process may even acquire help from effector CD8 T cells.^484–486^

#### Duration of immune response

Ideally, long-lasting protective efficacy would facilitate the eradication of pathogens and ease the medical and economic burden, especially for developing countries. Single dose IM-delivered VSV vectored vaccine has shown potential. As reviewed, 100% and 89% of participants remained seropositive at 2 years after a single high or low dose of rVSV-ZEBOV vaccination, respectively. NAbs were less durable, with seropositivity falling from 64–71% at 28 days to 27–31% at 6 months.^94^ Likewise, VSV vector vaccine achieved a long-lasting immune response in other hemorrhagic fever viruses.^101,102^ VSVΔG/LASVGPC induced rapid and long-term immunity to LASV. Post a single IM dose vaccination in guinea pigs, the protection rate was 100%, 87%, 83% and 71% on day 14, day 25 day 6 months and day 1 year, respectively.^102^ Further, the persistence of IN or OR- delivered VSV vector vaccine should be assessed.

In general, RABV-vector vaccines are designed as inactivated or OR delivered, while PIV vector vaccine are IN delivered. Diversity in immunization programs hindered the point-to-point comparison between these viral vectors. Mice orally inoculated with a single dose of rERAG333E produced strong and one year-long NAbs to RABV. 100% of vaccinated animals were protected from challenge of RABV at 12 months after immunization. Dogs who received one or two OR vaccinations with rERAG333E generated a strong protective NAbs response lasting for over 3 years, and moderate saliva RABV-specific IgA was also detected.^335^ In the case of PIV-vectored COVID-19 vaccines, the duration of the immune response after one or two IN doses of CVXGA vaccination in hamsters was measured and compared with those of two doses of COVID-19 mRNA vaccines. At day 36 post vaccination, 2X mRNA induced the upmost level of anti-S ELISA titers, 2X CVXGA1-immunized hamsters induced higher anti-S titers than 1X CVXGA1 immunized hamsters. Interestingly, anti-S titers on day 108 were comparable for all three vaccination groups. Compared to mRNA vaccines, anti-S ELISA titers and NAb titers in CVXGA1 vaccination groups were well maintained. The animal challenge study confirmed this phenomenon.^164^ When hamsters were challenged at 9 months post vaccination, CVXGA1 immunized hamsters were well protected than mRNA vaccine. The live-attenuated MeV vaccine has also been proven to elicit long-lasting B-cell and T-cell responses, with a reported measles-specific antibody half-life of more than 200 years.^487^ Duration in protective immune response could be attributed to the prolonged replication and spread of MeV in lymphoid tissue.^488^

Long-lasting protective efficacy was also observed in IFV-vectored COVID-19 vaccines. For example, dNS1-RBD induced a protective immune response lasting at least one year in hamster models.^235^ The above results indicate that the replication-competent viral vector vaccine exhibits an excellent profile in the persistence of protective immunity against multiple pathogens despite different delivery routes.

In contrast, AdV and poxvirus vector vaccines are largely designed as single-round replication or replication-defective constructs. Although the single-dose regimen of these vaccines has been tested in human clinical trials or approved. Less well immune persistence was observed when standing alone or applied as a single-dose regime compared to those replication-competent viral vector constructs.^95,248^ Representatively, in the Phase II clinical trial of ChAd3-EBO-Z and rVSV∆G-ZEBOV-GP in Liberia, seroconversion of the ChAd3-EBO-Z vaccination group was 63.5% at 1 year post a single IN dose vaccination, which was lower than the 79.5% rVSV∆G-ZEBOV-GP vaccination group.^262^ Generally, a prime-boost strategy was conducted to prolong the persistence of the immune response.^262,285,286^

#### Overcome the anti-vector immunity

Preexisting anti-vector immunity is a common problem faced by all viral vector vaccines, especially in AdV vectored-vaccines.^251,252,489–491^ Relatively, NNSVs and were less dampened by anti-vector immunity due to their single dose regimen, low serum positive rate, or replacement of surface glycoproteins.^155,193,205,206,492,493^ However, Serum positive rates of AdV are prevalent worldwide, ranging from 58.4 to 90%.^342,494–502^ Current solutions include increasing doses, selecting vectors with low seropositivity, chenge of delivery route and heterologous prime-boost strategy. The above solutions reduced the impact of anti-vector immunity to some extent. To fundamentally overcome this issue, the novel solution involves an immune escape strategy that deletes or modifies relevant regions, sequences, or epitopes of the viral vector targeted by pre-existing immunity. For example, the major determinants of AdV neutralization are in the fiber and hypervariable regions (HVRs) of Hexon protein, while replacing of seven short hypervariable regions on the surface of Ad5 hexon protein with corresponding HVRs of rare AdV serotype Ad48 successfully bypassed anti-Ad5 immunity.^503^ In the future, the determination of the dominant site of anti-vector immunity and corresponding genome modification-based immune escape strategies should be conducted to address the issue of anti vector immunity.

#### Advantages, limitations, and potential entry points

NNSV vectors share several advantages. The RNA of NNSV is not likely to integrate into the host genome and thus recombination rarely occurs.^35,504^ Besides, NNSV can quickly grow to high titers and propagate in appropriate cell lines, facilitating large-scale production. Meanwhile, the genome of NNSV is simple and easy-operated, thus the insertion of one or more foreign antigens and the rescue of recombinant virus is convenient. The NNSV genome harboring a foreign gene is relatively stable. It does not have issues with genome recombination and loss of foreign genes as frequently happens with positive-stand RNA virus genomes.^505,506^ Generally, low seropositive rate was reported in NNSV, and replacement of glycoprotein could further alleviate anti-vector immunity. Regarding and steps taken to address the paramount challenge of these NNSV vectors, the biosafety issue, was reviewed in part 3.2.

There are 18 subtypes of IFV HA and 11 subtypes of NA. By replacing HA and NA, chimeric viruses can be rescued through reverse genetics. Currently, the IFV vaccine production platform is highly optimized, permitting large-scale manufacturing.^231^ Nevertheless, capacity limitations may hinder the full application of IFV, as the length of foreign gene insertion is limited to about 1.5 kb nucleotides or less. What’s more, transgene stability of IFV-vectored vaccine should be improved. Recently, A/PR/8/1934 (H1N1) (PR8) and A/WSN/33 (H1N1) (WSN) are the most frequently used IFV skeletons. Although both of them are of low pathogenicity and can be handled in biosafety level two (BSL-2) laboratories, IN immunization may cause reassortment with circulating strains, leaving safety concerns. Changing the delivery route may avoid the reassortment. Additional studies concerning the potential mechanism to overcome species-specific restriction of IFV are needed to address the issue of reassortment in influenza.^507^

AdVs are well established viral vectors that have been fully evaluated in human clinical trials. These single-round replicated recombinant viruses are safe and well tolerated in humans. Subsequently, the immunogenicity and duration of these vaccines warrant further optimization.

Poxvirus vectors have some unique properties.^378^ (1) Lack of genomic integration in the host due to their cytoplasmic replication. (2) Low prevalence of anti-vector immunit. (3) Acceptable safety profiles in humans, particularly for ALVAC and MVA, their inability to replicate in mammalian cells further underlies their improved safety profile. (4) Established procedures for the large-scale production of clinical grade material. Compared to NNSV or AdV vectors, poxvirus appears to be less immunogenic in the application of prophylactic vaccines against viral haemorrhagic fever or beta coronavirus, as clinical trials of poxvirus vector vaccines are largely combined with other vaccine platforms. Seeking to optimize the poxvirus vector, several strategies have been implemented, including heterologous prime/boost protocols, use of co-stimulatory molecules, deletion of viral immunomodulatory genes still present in the poxvirus genome, enhancement of virus promoter strength, enhancement of vector replication capacity, optimizing expression of foreign heterologous sequences, and the combined use of adjuvants.^508^

## Conclusion and perspective

In response to acute public health events, viral vector vaccine platforms facilitate a timely response. Notably, viral-vectored COVID-19 vaccines achieved remarkable progress in human clinical trials and have been approved in a short period of time during the pandemic of COVID-19. Although potent immune response and protective efficacy have been conferred, intrinsic properties and extraordinary superiorities of these viral-vectored vaccines have not been fully exploited, especially mucosal delivery and mucosal immunity. Also seen in therapeutic cancer vaccines, advances in viral vector vaccines rely on improved understanding of viral biology and updated insights into reciprocal interactions between viruses and the host immune system.^509,510^

In the near future, a better understanding of the similarities and individualities of these viral vectors would push the revolutionary advances. Typically, NNSV is a large group of viral vectors that share collective viral biology characteristics and confronting homologous obstacles. Indeed, some progress has been made owing to the comprehensive knowledge of these NNSVs, including reverse genetic approaches, polarized transcription mechanism, chimeric strategy that retained the TMCT origin for foreign antigen incorporation, as well as the attenuation modification. Particularly important, the trained immunity induced by respiratory virus offer substantial benefits for antimicrobial infections and anti-tumor activity.^511–513^ Nevertheless, far more aspects should be taken into consideration under the in-depth master of similarities between viral vectors. Correspondingly, issues like anti-vector immunity, and safety concerns could be addressed in a same manner. Further, the individualities of these viral vectors should be clearly elucidated depending on the targeted pathogens or neoplasm. In this process, interdisciplinary cooperations, structural biology, artificial intelligence and gene editing, etc. may provide additional support.

As has been exhaustively reviewed, VSV and MeV are distinguished viral vectors and of the potential for mucosal delivery and induction of durable local and systematic immune response. Nevertheless, VSV and MeV-based COVID-19 initiated by Merck, V590^332^ and V591,^198,199^ received disappointed responses in Phase I clinical trials despite promising results in preclinical studies (Table 10). This could be attributed to the less well connection and coordination between preclinical and clinical trials, specifically, the suboptimal selection of delivery route. Ideally, animal models should accurately and comprehensively reflect the immune status and post-vaccination response in human beings, conversely, outcomes from inappropriate animal models would mislead the experimental design of the clinical trial, ultimately determining the final direction. For VSV and MeV, their potential for mucosal delivery were largely unexplored, particular in clinical trials. Consequently, the essential attributes of these NSSVs warrant further investigation in human clinical trials on the basis that convincing approaches achieved in preclinical trials. Overall, appropriate and accurate technological advances, sufficient exploration in potential, and tightly connection between preclinical and clinical studies would consolidate the position of viral vector vaccines and to compel the acceleration and approval of novel viral vector vaccines.

**Table 10:** Viral vectored vaccines in clinical trials

Vector	Pathogens	Developer	Constructs (name)	Reported status	Results	Clinical trials registry
VSV	EBOV	Merck	VSV-ZEBOV-G(Ervebo)	Phase III	An overall protective efficacy of 100%	PACTR201503001057193
		Profectus	N4CT1-GP1	Phase I	Safe and immunogenic	NCT02718469
	SARS-CoV-2	Merck	rVSVΔG-S (V590)	Phase I	Poor immunogenicity	NCT04569786
PIV	RSV	AstraZeneca	B/HPIV3-F	Phase I	Safe and immunogenic in infants and children	NCT00493285 NCT00345670
MeV	CHIKV	Rostock University	MV-CHIKV VLP	Phase II	Well-tolerated; immunogenic; persistent in immune response	NCT02861586
	SARS-CoV-2	Merck	MeV-S (V591)	Phase I/II	Well tolerated but insufficient immunogenicity	NCT04498247
NDV	SARS-CoV-2	Mahidol University	NDV-S(NDV-HXP-S)	Phase I	Safe and immunogenic	NCT04764422 NCT04871737
	Cancer	Israel	NDV-HUJ	Phase I/II	Good tolerability and encouraging responses	\
IFV	SARS-CoV-2	Wantai BioPharm	dNS1-RBD	Phase III	55% and 82% protection for people without/with immunization history	ChiCTR2100051391
	IFV	AstraZeneca	FluMist	\	78–100% protection against different IFV strains	\
AdV	EBOV	CanSino	Ad5-Makona GP	Phase II	Safe and highly immunogenic; 8 × 10^10^ viral particles was validated as the optimal dose	PACTR201509001259869
		Russia	GamEvac-Combi	Phase II	100% seroconversion rate; robust immune response	0373100043215000055
		NIH	ChAd3-EBO-Z	Phase III/II	Immune responses largely maintained through 12 months	NCT02344407
	SARS-CoV-2	CanSino	Ad5-S (Convidecia)	Phase III/IV	57.5% efficacy against symptomatic; heterologous boosting with Convidecia following with inactivated COVID-19 vaccine is safe and more immunogenic than homologous prime boost	NCT04526990 NCT04892459
			Ad5-S (Convidecia Air)	Phase III	Heterogenous boost with inactivated vaccine induced better immune response than homologous prime boost	NCT05043259
		Janssen	Ad26-S (Jcovden)	Phase III	52.9% protection against symptomatic infection	NCT04505722
		Gamaleya	Ad5 + Ad26-S (Sputnik V)	Phase III	91.6% overall efficacy	NCT04530396
			Ad26-S (Sputnik Light)	Phase I	Safe and immunogenic	NCT04741061
		AstraZeneca	ChAdOx1-S (Vaxzevria)	Phase III	70.4% overall efficacy	NCT04324606, NCT04444674
			Vaxzevria (i.n.)	Phase I	Tolerate, mucosal and systemic response	NCT04871737
		Vaxart	Ad5-S + N(oral tablet)	Phase I	Safe and generated mucosal immune responses	NCT04563702
	MERS-CoV	Saudi Arabia	ChAdOx1 (MERS002)	Phase I	Safe and immunogenic	NCT04170829
	RABV	Oxford	ChAdOx2 RabG	Phase I	Safety, tolerate and immunogenic	NCT04162600
	Cancer	Spain	DNX-2401	Phase I	Dramatic responses with long-term survival in gliomas	NCT00805376
		Sunway	Oncorine (H101)	Phase III	FDA approved for head and neck neoplasms	\
		FerGene	Nadofaragene firadenovec	Phase III	Efficacious and favorable benefit	NCT02773849
Pox virus	EBOV	Janssen	Ad26-ZaireGP+MVA-BN-Filo	Phase I	Well tolerated; highly immunogenic; long-lasting antibodies duration (1 year)	NCT02376426
		GlaxoSmithKline	ChAd3-ZaireGP+MVA-BN-Filo	Phase I	Safe and immunogenic	NCT02231866 NCT02267109
	MERS-CoV	Germany	MVA- S	Phase I	Safe and immunogenic	NCT03615911
	HIV	USA	ALVAC-HIV + protein vaccine	Phase III	Controversial efficacy	NCT02404311 NCT02968849
	Smallpox	Bavarian Nordic	MVA	Phase III	Safe, seroconversion rate over 90.8%	NCT01913353
HSV-1	Cancer	University of T okyo	HSV-1	Phase II	Survival benefit and safety profile	NCT02457845 UMIN000015995
		Amgen	HSV-1	Phase III	Well tolerated, longer durable response rate and longer survival	NCT00769704

*VSV* vesicular stomatitis virus, *PIV* parainfluenza virus, *MeV* measles virus, *NDV* Newcastle disease virus, *IFV* influenza virus, *AdV* adenovirus, *EBOV* Ebola virus, *SARS-CoV-2* severe acute respiratory syndrome coronavirus 2*, RSV* respiratory syncytial virus, *MERS-CoV* Middle East respiratory syndrome coronavirus, *RABV* rabies virus, *HIV* Human immunodeficiency virus, *HSV-1* Herpesvirus type I
